# The composition of environmental microbiota in three tree fruit packing facilities changed over seasons and contained taxa indicative of *L. monocytogenes* contamination

**DOI:** 10.1186/s40168-023-01544-8

**Published:** 2023-06-05

**Authors:** M. Laura Rolon, Xiaoqing Tan, Taejung Chung, Narjol Gonzalez-Escalona, Yi Chen, Dumitru Macarisin, Luke F. LaBorde, Jasna Kovac

**Affiliations:** 1 Department of Food Science, The Pennsylvania State University, University Park, PA 16802 USA; 2 Microbiome Center, The Pennsylvania State University, University Park, PA 16802 USA; 3 Center for Food Safety and Applied Nutrition, Food and Drug Administration, College Park, MD 20740 USA

**Keywords:** Microbiota, *Listeria monocytogenes*, Food safety, Spatial and temporal variation, Tree fruit packing facilities

## Abstract

**Background:**

*Listeria monocytogenes *can survive in cold and wet environments, such as tree fruit packing facilities and it has been implicated in outbreaks and recalls of tree fruit products. However, little is known about microbiota that co-occurs with *L. monocytogenes* and its stability over seasons in tree fruit packing environments. In this 2-year longitudinal study, we aimed to characterize spatial and seasonal changes in microbiota composition and identify taxa indicative of *L. monocytogenes* contamination in wet processing areas of three tree fruit packing facilities (F1, F2, F3).

**Methods:**

A total of 189 samples were collected during two apple packing seasons from floors under the washing, drying, and waxing areas. The presence of *L. monocytogenes* was determined using a standard culturing method, and environmental microbiota was characterized using amplicon sequencing. PERMANOVA was used to compare microbiota composition among facilities over two seasons, and abundance-occupancy analysis was used to identify shared and temporal core microbiota. Differential abundance analysis and random forest were applied to detect taxa indicative of *L. monocytogenes* contamination. Lastly, three *L. monocytogenes*-positive samples were sequenced using shotgun metagenomics with Nanopore MinION, as a proof-of-concept for direct detection of *L. monocytogenes*’ DNA in environmental samples.

**Results:**

The occurrence of *L. monocytogenes* significantly increased from 28% in year 1 to 46% in year 2 in F1, and from 41% in year 1 to 92% in year 2 in F3, while all samples collected from F2 were *L. monocytogenes*-positive in both years. Samples collected from three facilities had a significantly different microbiota composition in both years, but the composition of each facility changed over years. A subset of bacterial taxa including *Pseudomonas*, *Stenotrophomonas*, and *Microbacterium*, and fungal taxa, including *Yarrowia*, *Kurtzmaniella*, *Cystobasidium*, *Paraphoma*, and *Cutaneotrichosporon*, were identified as potential indicators of *L. monocytogenes* within the monitored environments. Lastly, the DNA of *L. monocytogenes* was detected through direct Nanopore sequencing of metagenomic DNA extracted from environmental samples.

**Conclusions:**

This study demonstrated that a cross-sectional sampling strategy may not accurately reflect the representative microbiota of food processing facilities. Our findings also suggest that specific microorganisms are indicative of *L. monocytogenes*, warranting further investigation of their role in the survival and persistence of *L. monocytogenes*.

Video Abstract

**Supplementary Information:**

The online version contains supplementary material available at 10.1186/s40168-023-01544-8.

## Introduction

*Listeria monocytogenes*, the causative agent of human listeriosis, is a foodborne pathogen that is particularly threatening to susceptible populations, including pregnant women and their newborns, the elderly, and the immunocompromised [1]. In the USA, foodborne listeriosis has been estimated to have a 94% rate of hospitalization and 15.9% death rate [2]. In recent years, outbreaks of listeriosis have been linked with the consumption of whole fruits, such as apples, which have previously not been considered high-risk foods due to their low pH [3–5]. However, the 2014 outbreak of *L. monocytogenes* that was traced back to commercially produced prepackaged caramel apples [3] triggered extensive monitoring of tree fruit packing environments to assess the prevalence of *L. monocytogenes* contamination [6].

*Listeria* is commonly found in orchard environments, especially in soil and decaying vegetation [7] and can therefore be introduced to tree fruit packing environment with produce, together with other soil- and plant-associated microbiota [8]. Once in the tree fruit packing environment, the microbiota composition of a facility is shaped by building design factors, the immediate outdoor environment, as well as its human occupants [9]. Food processing facilities are a unique category among built environments, where the processes carried out within a facility necessitate the recurrent introduction of raw food ingredients. This allows for an influx of microbial communities native to the farm or natural environment as well as nutrients that facilitate their growth. Unlike many other built environments, the microbiota residing in food processing environments is subjected to recurrent antimicrobial pressures due to daily cleaning and sanitizing operations aimed to reduce the environmental microbial load [8, 10]. However, due to high humidity, availability of organic matter, and the ability of some microbiota to grow at low temperatures, microorganisms that survive antimicrobial treatments may grow and colonize such environments [11]. *L. monocytogenes* has been shown to persist in food processing environments over long periods of time by inhabiting spaces that are challenging to clean and sanitize [12]. Persistent colonization of facilities by *L. monocytogenes* leads to increased risk for recurrent contamination of food [13]. Hence, application of effective cleaning and sanitizing protocols is of critical importance for control of *L. monocytogenes*. The effectiveness of cleaning and sanitizing of food processing environments can be challenged by the ability of some environmental microorganisms to form multispecies biofilms that can facilitate the survival of *L. monocytogenes.* For example, it has been shown that co-culturing *Listeria monocytogenes* with strains of *Pseudomonas* spp. results in the formation of more robust biofilms compared to those produced by *Listeria monocytogenes* alone [14, 15]. While the nature of *Pseudomonas-Listeria* interactions has been previously characterized, there is limited knowledge available on the potential interactions between *L. monocytogenes* and other food processing environment microbiota that may be of food safety relevance.

Despite the need for better understanding of microbial residents in fresh produce processing environments, the characterization of microbiota in food processing environments has largely been limited to facilities that process fermented foods [16–29]. These studies have mainly focused on identifying the taxonomy of bacteria and fungi present in food processing environments and its association with the fermentation processes of cheese [16, 20–24, 26], sauerkraut [28], alcoholic beverages [18, 19, 30, 31], and sourdough [29]. Some also attempted to use microbiota characterization to track the sources of spoilage organisms of processed meats [27, 32, 33] and ready-to-eat meals [27], and to track changes in the microbiota after cleaning and sanitizing in slaughterhouses [34]. To the best of our knowledge, only two studies surveyed microbiota in fresh produce processing facilities [10, 35]. In one of these studies, we previously monitored bacterial and fungal microbiota composition, as well as *L. monocytogenes* presence in three tree fruit packing facilities over one apple packing season (November 2017–April 2018) [35]. Here, we report findings from microbiota and *L. monocytogenes* monitoring in a subsequent apple packing season (2018–2019) that was conducted to (i) evaluate seasonal variability in the composition of environmental microbiota and occurrence of *L. monocytogenes*, (ii) to identify the common, temporal, and ecological core microbiota of studied environments [36], and (iii) to identify microbial taxa indicative of *L. monocytogenes* contamination. In addition to these aims, we used Nanopore MinION for shotgun sequencing of three collected samples as a proof-of-concept for direct detection of *L. monocytogenes*’ DNA in environmental samples.

## Methods

### Study design and sample collection

Three tree fruit packing facilities (F1, F2, and F3) located in the northeast of the USA, previously described by Simonetti et. al [6], were sampled for the duration of an apple packing season (November 2018 through February 2019) to assess the occurrence of *L. monocytogenes* and the composition of the environmental microbiota. To evaluate seasonal variability in microbiota composition and *L. monocytogenes* occurrence, samples that had been previously collected between November 2017 and April 2018 and reported in Tan et al. [35] were included for comparative data analyses in this study. We refer to samples reported in Tan et al. [35] as samples collected in season 1 (i.e., year 1 [Y1]: November 2017–April 2018) and samples collected within this study as samples from season 2 (i.e., year 2 [Y2]: November 2018–February 2019). Samples from both years were collected at the same three locations within the same three tree fruit packing facilities.

**Fig. 1:**
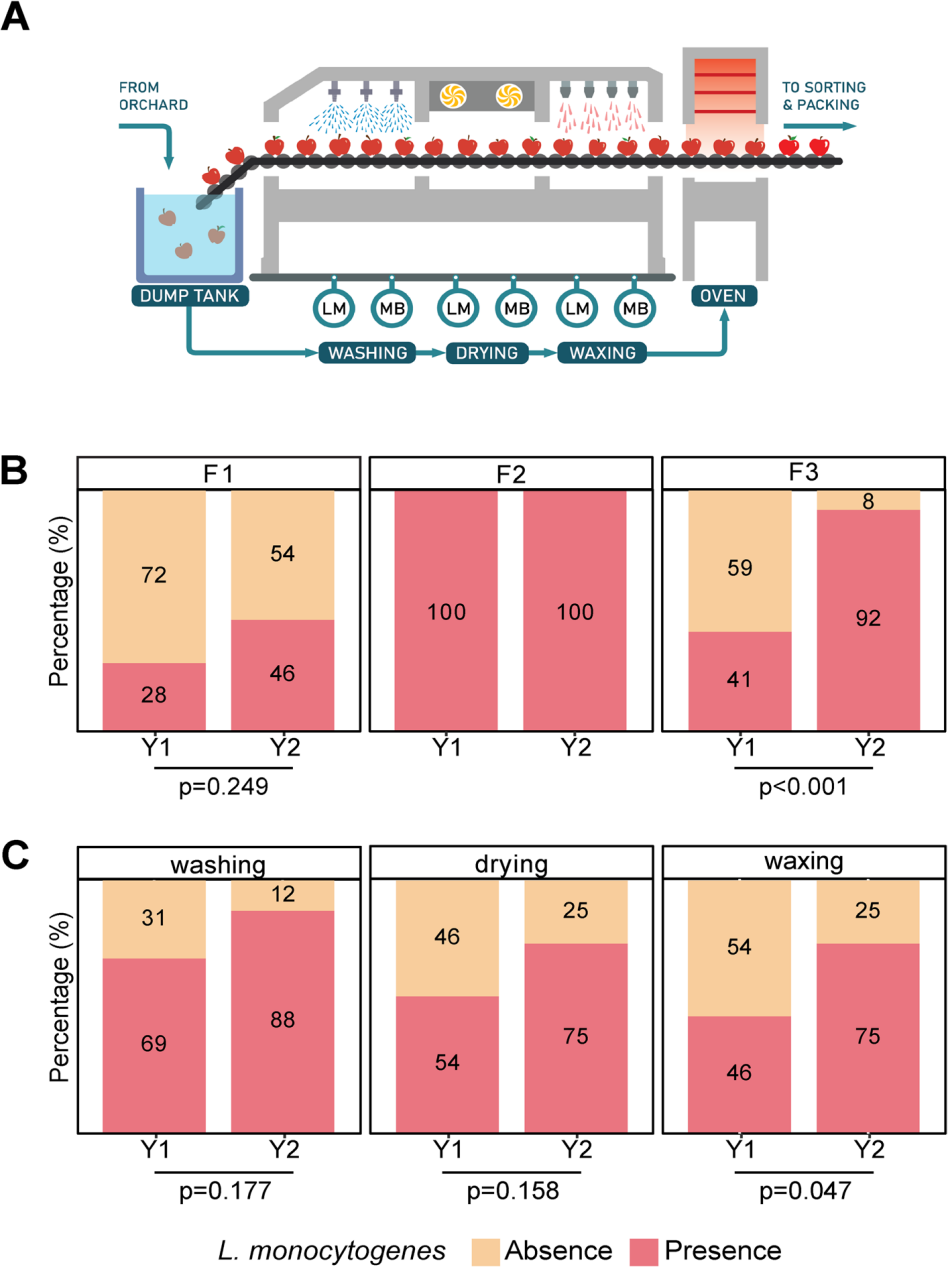
Prevalence of* L. monocytogenes *in tree fruit packing facilities throughout two packing seasons. Environmental samples were collected from the floor under the brush conveyor belt where washing, fan-drying, and waxing processes are carried out in three tree fruit packing facilities (F1, F2, F2) (**A**). The prevalence of *L. monocytogenes* is shown by facility (**B**) and by processing section (**C**) during sampling conducted in two seasons: year 1 (Y1) and year 2 (Y2). A total of 39 and 24 samples were collected per facility in Y1 and Y2, respectively. The presence and absence of viable *L. monocytogenes*, as determined by an enrichment method, is shown in pink and orange, respectively. The *p*-values denote significant differences between samples collected in Y1 and Y2 using a two-proportion *Z*-test to statistically evaluate the differences in the occurrence of *L. monocytogenes*

Environmental samples were collected from non-food contact surfaces located in zone 3 (i.e., non-food contact surfaces within the facility that are not in close proximity to food contact surfaces, e.g., floor, drains) [37, 38]. On each sampling date, two samples (i.e., biological replicates) were collected from adjacent sites underneath each of the three sections of a conveyor belt that transported fruit through the washing, fan-drying, and waxing operations (Fig. 1A). One of the biological replicates was used for *L. monocytogenes* detection, and the second one was used for microbiota characterization. Samples for *L. monocytogenes* detection were collected with sponges hydrated with 10 mL D/E Neutralizing Buffer (3 M, HS10DE2G, Saint Paul, MN) to enhance the survival of *Listeria* through neutralization of potential sanitizer residues. Samples for microbiota characterization were collected using sponges hydrated with 10 mL of Neutralizing Buffer (3 M, HS2410NB2G, Saint Paul, MN). All samples were collected by swabbing a 40 cm by 40 cm area (10 times horizontally and 10 times vertically). Samples were stored in a cooler with ice packs during transportation to the laboratory and were processed on the day of the collection. A total of 117 samples had been collected in Y1, and 72 samples were collected in Y2.

### *Listeria* spp. isolation and *L. monocytogenes* identification

Detection of *Listeria* was conducted by following the FDA BAM protocol “Detection and Enumeration of *Listeria monocytogenes* in Foods and Environmental Samples” [39]. Briefly, 90 ml of Buffered *Listeria* Enrichment Broth (BLEB) were added to each test sample, as well as to two positive controls (*L. monocytogenes* strain F2365 = PS00838 [40] and *L. innocua* strain PS00298), and a negative control (a sterile sampling sponge). Samples were manually homogenized by hand-massaging the sponge 15 times, followed by incubation for 4 h at 30 °C to facilitate the recovery of injured cells. After incubation, 400 μl of BLEB supplement (90 mg acriflavine HCl, 360 mg nalidixic acid, and 450 mg cycloheximide in 40 ml of sterile water) (Sigma Aldrich, St. Louis, MO) was added to each test sample and controls. Samples were then further incubated for 44 ± 2 h at 30 °C. After incubation, a loopful of each enrichment was streaked onto Agar Listeria Ottavani & Agosti (ALOA) (BioRad Laboratories Inc., Hercules, CA) and RAPID’ L mono (BioRad Laboratories Inc., Hercules, CA) selective differential agars and incubated for 48 h at 37 °C. One isolated presumptive *L. monocytogenes*-positive colony was selected from each selective differential agar, re-suspended in nuclease-free water, and heated in a water bath for 10 min at 95 °C to lyse the cells. Cell lysates were then centrifuged at 15,000* g* for 10 min to pellet cell debris. Two microliters of the supernatant were used as a DNA template for species confirmation by multiplex PCR with primers targeting genes *iap* (specific for *Listeria* spp.) and *lmo2234* (specific for *L. monocytogenes*) [40]. The following thermal cycling conditions were used: initial denaturation at 95 °C for 15 min, 15 cycles of denaturation at 94 °C for 1 min, annealing at 55 °C to 51 °C for 1 min with a touch down of 3 cycles per temperature, extension at 72 °C for 1 min. The subsequent 15 cycles started with the denaturation at 94 °C for 1 min, annealing at 50 °C for 1 min, extension at 72 °C for 1 min and the final extension at 72 °C for 8 min [40]. *L. monocytogenes* and *L. innocua* strains served as positive controls and nuclease-free water was used as a negative control. Successful PCR amplification was confirmed by gel electrophoresis using a 2% agarose gel (Invitrogen, Carlsbad, CA). Specifically, a band at 1450 to 1600 bp (specific for *iap* gene of *Listeria* spp.) and a band at 420 bp (specific for *lmo2234* gene of *L. monocytogenes*) were expected in samples positive for *Listeria* spp. and *L. monocytogenes*, respectively.

Statistical analyses were conducted in R version 4.1.2 [41]. A chi-square test was carried out to assess the significance of differences in *L. monocytogenes* occurrence in samples collected from different facilities and processing line sections. Statistical analyses were carried out separately for each season, using the R package MASS (version 7.3.56) [42]. Fisher’s pairwise comparison of proportions test with a Bonferroni correction for multiple comparisons was carried out using the R package fmsb (version 0.7.3) [43] to assess statistical significance (*α* = 0.05). A two-proportion *Z*-test was conducted for each data pair to statistically evaluate the differences in the occurrence of *L. monocytogenes* in samples collected in Y1 and Y2.

### DNA extraction for bacterial and fungal microbiota sequencing

Fifty milliliters of a phosphate buffer containing 0.9% NaCl was added to each sample collected for microbiota characterization, and bags were stomached for 7 min at 230 rpm. The homogenate was transferred to a sterile conical tube and centrifuged at 11,000* g* and 4 °C for 20 min to pellet the biomass [44]. After centrifugation, the supernatants were discarded, and pellets were stored at − 80 °C until DNA extraction. DNA was extracted from the pellets using DNeasy PowerSoil DNA extraction kit (Qiagen, Hilden, Germany) by following the manufacturer’s protocol. Four negative controls were included at the DNA extraction step to control for the potential presence of DNA in the extraction kits or contamination during DNA extraction. After DNA extraction, the concentration of DNA in each sample was determined with Nanodrop One (Thermo Fisher Scientific, Waltman, MA) and Qubit 3 using Qubit dsDNA High Sensitivity (HS) Assay Kit (Thermo Fisher Scientific, Waltman, MA). DNA samples were stored at − 80 °C until PCR amplification, library preparation, and sequencing.

### PCR amplification

Bacterial and fungal microbiota composition was determined by sequencing of the PCR-amplified V4 domain of the 16S rRNA gene and the internal transcribed spacer 2 sequence (ITS2), respectively. Briefly, V4 region of the 16S rRNA gene sequence was amplified using the forward primer 505F and the reverse primer 806R [45]. PCR reactions for 16S rRNA V4 region contained 12.5 μl of 2 × KAPA HIFI HotStart Ready Mix (Roche, Basel, Switzerland), 1 μl of 10 μM of each primer (IDT, Coralville, IA), 8.5 μl of nuclease-free water, and 2 μl of extracted DNA. PCR amplification included initial denaturation at 95 °C for 2 min, 25 cycles of denaturation at 98 °C for 20 s, annealing at 56.5 °C for 20 s, extension at 72 °C for 25 s, and the final extension at 72 °C for 5 min; final hold was at 4 °C. Fungal ITS2 sequences were amplified using the forward primer ITS4F and the reverse primer ITS9R [46]. PCR reactions for amplification of the ITS2 region contained 12.5 μl 2 × KAPA HIFI HotStart Ready Mix (Roche, Basel, Switzerland), 1 μl of 10 μM of each primer (IDT, Coralville, IA), 9.5 μl of nuclease-free water, and 1 μl of extracted DNA. PCR amplification included initial denaturation at 98 °C for 5 min, 35 cycles of denaturation at 95 °C for 30 s, annealing at 50 °C for 60 s, extension at 72 °C for 60 s, and final extension at 72 °C for 5 min; final hold was at 4 °C. PCR amplicons were visualized by gel electrophoresis (130 V for 30 min) using a 2% agarose gel stained with SYBR Safe (Thermo Fisher Scientific, Waltman, MA) to confirm successful amplification.

### 16S rRNA V4 and ITS2 amplicon library preparation and sequencing

Amplicon libraries were prepared based on Illumina’s 16S Metagenomic Sequencing Library Preparation protocol [47]. 16S rRNA V4 and ITS2 PCR products were barcoded with unique combinations of i7 and i5 index adaptors (IDT, Coralville, IO) in a second-step PCR, including thermal cycling steps of initial denaturation at 95 °C for 3 min, 8 cycles of denaturation at 95 °C for 30 s, annealing at 55 °C for 30 s, extension at 72 °C for 30 s, and final extension at 72 °C for 5 min; final hold was at 4 °C. Barcoded libraries were purified with AMPure XP beads (Beckman Coulter, Pasadena, CA, USA), and DNA concentrations were normalized using Mag-Bind EquiPure Library Normalization Kit (Omega Bio-Tek, Norcross, GA, USA) following the manufacturer’s protocols. Concentrations of normalized libraries were verified using High Sensitivity dsDNA Assay kit (Thermo Fisher Scientific, Waltman, MA) with Qubit 3 fluorometer (Thermo Fisher Scientific, Waltman, MA), and the distribution of library fragment sizes was measured with Bioanalyzer (Agilent, Santa Clara, CA). Libraries were then diluted to 4 nM and pooled in equal volumes of 4 μl for each sample. The final concentration of the pooled library was verified using the High Sensitivity dsDNA Assay kit with a Qubit 3 fluorometer (Thermo Fisher Scientific, Waltman, MA). The pooled library was denatured by mixing 5 μl of 4 nM pooled library with 5 μl freshly prepared 0.2 N NaOH and further diluted with a pre-chilled HT1 buffer (Illumina, San Diego, CA) to 7.5 pM. PhiX (10%) was added as an internal control library. A total of 600 μl of denatured library was loaded onto an Illumina MiSeq (Illumina, Inc., San Diego, CA) flow cell. Two sequencing runs were performed using 500 cycle V2 Illumina sequencing kit (Illumina, San Diego, CA) for 250 bp paired end sequencing, and both 16S rRNA V4 and ITS libraries were included in each sequencing run.

### Bioinformatic analyses

To allow for comparison of results obtained for samples collected in two sampling seasons, sequences obtained in Y1 previously reported in Tan et al. [35] were reanalyzed in conjunction with sequences for samples collected in Y2, using a workflow described here. Raw sequences were analyzed using DADA2 (v. 1.22.0) [48]. Briefly, low-quality paired end sequencing reads were removed and trimmed to 200 bases for forward and reverse reads, respectively. The error rates were estimated from the reads, followed by inference of Amplicon Sequence Variants (ASVs) and merging of paired end reads. Chimeras were detected and removed, and taxonomy was assigned to the ASVs by alignment against the reference database Silva v132 [49] for 16S rRNA V4 sequences and UNITE v8 [50] for ITS2 sequences. Prior to subsequent statistical analyses, ASVs assigned to chloroplast and mitochondria were removed from the dataset.

### Statistical analysis of bacterial and fungal microbiota composition

We used a compositional data analysis framework [51, 52] to characterize the bacterial and fungal microbiota of the three tree fruit packing facilities. All ASVs with “0” count values were assigned a small non-zero value using the Count Zero Multiplicative Method using the R package zCompositions v1.4.0–1 [53] prior to applying a center-log ratio (CLR) transformation. To determine whether there was a significant effect of the year or facility on the microbiota composition, Aitchison distances were calculated using CLR-transformed data, and Pairwise Permutational Multivariate Analysis of Variance (PERMANOVA) was used with a linear model that included the effects of facility, year, and their interaction, using the R package pairwiseAdonis v0.4 [54], with 999 permutations. *P*-values were corrected for multiple comparisons using the Bonferroni correction. To further investigate the differences in bacterial and fungal microbiota composition by facility within each season, the ASV tables for bacteria and fungi were split by year. Principal component analysis was performed on CLR-transformed data for each dataset (Bacteria Y1, Bacteria Y2, Fungi Y1, and Fungi Y2) to visualize differences in bacterial and fungal microbiota composition. Further, a PERMANOVA linear model was applied on the Aitchison distances of each dataset to assess the effect of each facility, presence of *L. monocytogenes*, and their interaction. To further investigate the differences in bacterial and fungal microbiota composition by season within each facility, the ASV tables for bacteria and fungi were split by facility. A PERMANOVA linear model was applied on the Aitchison distances of each dataset to identify the effect of year on the microbiota composition of each facility. To investigate the taxonomic composition of samples, relative abundances of taxa were calculated using the Aitchison Simplex method with the R package compositions v2.0–3 [55]. The composition of microbiota was visualized at the ASV level with bar plots and heatmaps using the package ggplot2 v3.3.6 [56].

To identify temporal core bacterial and fungal microbiota in each of the three fruit packing facilities and across all three facilities, abundance-occupancy distributions were calculated for each facility using the method described by Stopnisek and Shade [57]. In this study, we defined the temporal core bacterial and fungal microbiota of each individual facility as ASVs that were present in a facility in at least one out of the three samples collected on each sampling date, throughout two sampling seasons (i.e., ASVs with an occupancy of 1). We defined the common temporal core bacterial and fungal microbiota as the temporal core ASVs that were shared across all facilities. To further characterize the relationship between core taxa and the rest of the microbiota, network analysis was performed using the SPIEC-EASI method [58] implemented in the NetCoMi package (v1.0.2) [59]. To reduce the sparsity of the data, only bacterial and fungal ASVs that were present in at least 50% of the samples (i.e., occupancy = 0.5 to 1) [57] were included. Topological features of networks, including the edge number, diameter, transitivity, mean distance, betweenness centrality, and modularity, were calculated for the global network using the NetCoMi package v1.0.2 [59]. Network hubs were determined as the nodes with the highest betweenness centrality.

Differential abundance analysis was carried out to statistically assess differences in the composition of bacterial and fungal microbiota within each facility between two seasons. For this purpose, the R package ALDEx2 v1.26.0 [60] was used to identify differentially abundant ASVs using default parameters [52]. Similarly, ALDEx2 was used to determine whether samples (from all three facilities and both years combined) with detected viable *L. monocytogenes* had differentially abundant ASVs compared to those in which viable *L. monocytogenes* was not detected.

Random forest classification was used to identify ASVs that were indicative of the presence of *L. monocytogenes* in tree fruit packing environments. To build the model, ASV tables were first reduced to exclude taxa with less than 30 reads in the 2-year dataset to reduce computational intensity of the analysis, resulting in 15,514 and 1731 ASVs included in the model for bacteria and fungi, respectively. The ASVs and their relative abundances were used as variables for classification of samples into *L. monocytogenes*-positive and -negative categories. Ten-fold cross validation was repeated three times to tune two hyperparameters (i.e., mtry: number of variables included in each random subset for each splitting nodes, ntree: number of trees constructed in each run) to identify the optimal classification model based on the classification accuracy, using caret package v6.0–92 [61]. After completed tuning, the classification model was developed, and variable importance was calculated based on the mean decrease in accuracy of each ASV for the classification using randomForest v4.7–1.1 [62]. The model’s classification reliability was also assessed based on the area under the curve (AUC).

### Nanopore sequencing

Nanopore shotgun metagenomic sequencing was used as a proof-of-concept for the direct detection of *L. monocytogenes*’ DNA in environmental samples. Three samples that tested positive for *L. monocytogenes* using a standard culturing method were selected for sequencing using Nanopore MinION (Oxford Nanopore Technologies, Oxford, UK). The selected samples were collected on the same sampling day (February 21st, 2019) from the washing section of each facility (Table S1). DNA that had been previously extracted for amplicon sequencing was used to prepare libraries with the Genomic Ligation kit (SQK-LSK109, Oxford Nanopore Technologies, Oxford, UK). The three prepared libraries were sequenced using individual FLO-MIN106 (R9.4.1, Oxford Nanopore Technologies, Oxford, UK) flow cells with a MinION device for 48 h. Data were collected and called in real time using the MinKNOW software v19.10.1 (Oxford Nanopore Technologies, Oxford, UK). The What’s in My Pot (WIMP) v3.2.2 workflow available through the EPI2ME cloud service (Epi2me Desktop Agent, v19.09.23) was used for taxonomic classification of reads. Briefly, WIMP removed sequences with a mean q-score below 7 and used Centrifuge [63] to classify reads using a pre-built data structure based on the NCBI RefSeq database, which supported the identification of bacteria, viruses, fungi, and archaea.

## Results

### *L. monocytogenes* occurrence was high in all three monitored tree fruit packing facilities, and it significantly increased from first to second season in facility F3

Among samples collected in Y1, 11 (28%), 39 (100%), and 16 (41%) were positive for *L. monocytogenes* in F1, F2, and F3, respectively [35]. In Y2, the occurrence of *L. monocytogenes* increased to 46% and 92% in F1 and F3, respectively, and remained constant at 100% in F2 (Fig. 1B). The occurrence of *L. monocytogenes* among facilities was significantly different in Y1 [35] as well as Y2 (*p* = 1.50*10^−6^) (Table S2). Specifically, in Y1, the occurrence of *L. monocytogenes* in F2 was significantly different from that of F1 (*p* = 8.20 × 10^12^) and F3 (*p* = 6.6*10^−9^), while the occurrence did not significantly differ between F1 and F3 (*p* = 1.00). In Y2, the occurrence of *L. monocytogenes* in F1 significantly differed from that in F2 (*p* = 7.80 × 10^−5^) and F3 (*p* = 4.0 × 10^−3^). In contrast to the observation in Y1, the occurrence of *L. monocytogenes* was not significantly different in F2 compared to F3 in Y2 (*p* = 1.00). A two population proportions *Z*-test was used to statistically assess differences in the occurrence of *L. monocytogenes* in F1 and F3 between seasons (Table S3). F2 was not included in this analysis since all samples collected in this facility were positive for *L. monocytogenes* in both seasons. The occurrence of *L. monocytogenes* was not significantly different in F1 between the two sampling seasons (*p* = 0.25). However, the occurrence of *L. monocytogenes* in F3 significantly increased from Y1 to Y2 (*p* = 1.96 × 10^−4^).

Seasonal changes in the occurrence of *L. monocytogenes* were also assessed by the section of the tree fruit packing line (Fig. 1C). In the washing section, the occurrence of *L. monocytogenes* increased from 69% (*n* = 27) in Y1 to 88% (*n* = 21) in Y2 (Fig. 1C); however, this difference was not statistically significant (*p* = 0.18) (Table S3). In the fan-drying section, there was an increase in the occurrence of *L. monocytogenes* from 54% (*n* = 21) in Y1 to 75% (*n* = 18) in Y2; however, this increase was also not significant (*p* = 0.16) (Table S3). In contrast, in the waxing section, there was a statistically significant increase in the occurrence of *L. monocytogenes* from 46% (*n* = 18) in Y1 to 75% (*n* = 18) in Y2 (*p* = 0.047) (Table S3).

### The composition of environmental microbiota in tree fruit packing facilities was significantly different among facilities and between seasons

The 16S rRNA V4 and ITS2 regions were PCR amplified and sequenced for 117 samples in Y1 [35] and for 72 samples in Y2 to determine the composition of bacterial and fungal microbiota present in the three tree fruit packing facilities. None of the negative controls used to assess contamination during the DNA extraction step resulted in a positive amplification, thus these samples were not included in the library preparation or sequencing. Sequencing of 16S rRNA V4 gene amplicons resulted in a median of 59,464 and 32,530 reads, in Y1 and Y2, respectively. The difference in average 16S rRNA amplicon sequencing depth between Y1 and Y2 samples was not statistically significant (*p* = 0.280) (Fig. S1A). The sequencing depth of ITS2 amplicons was significantly higher (*p* < 2.2 × 10^−16^) in Y2 with a median of 91,701 reads, compared to Y1 with a median of 36,078 reads (Fig. S1B). Sequencing reads were processed using the DADA2 pipeline to obtain 36,671 and 7917 unique Amplicon Sequence Variants (ASVs) for the bacterial and fungal microbiota, respectively.

**Fig. 2:**
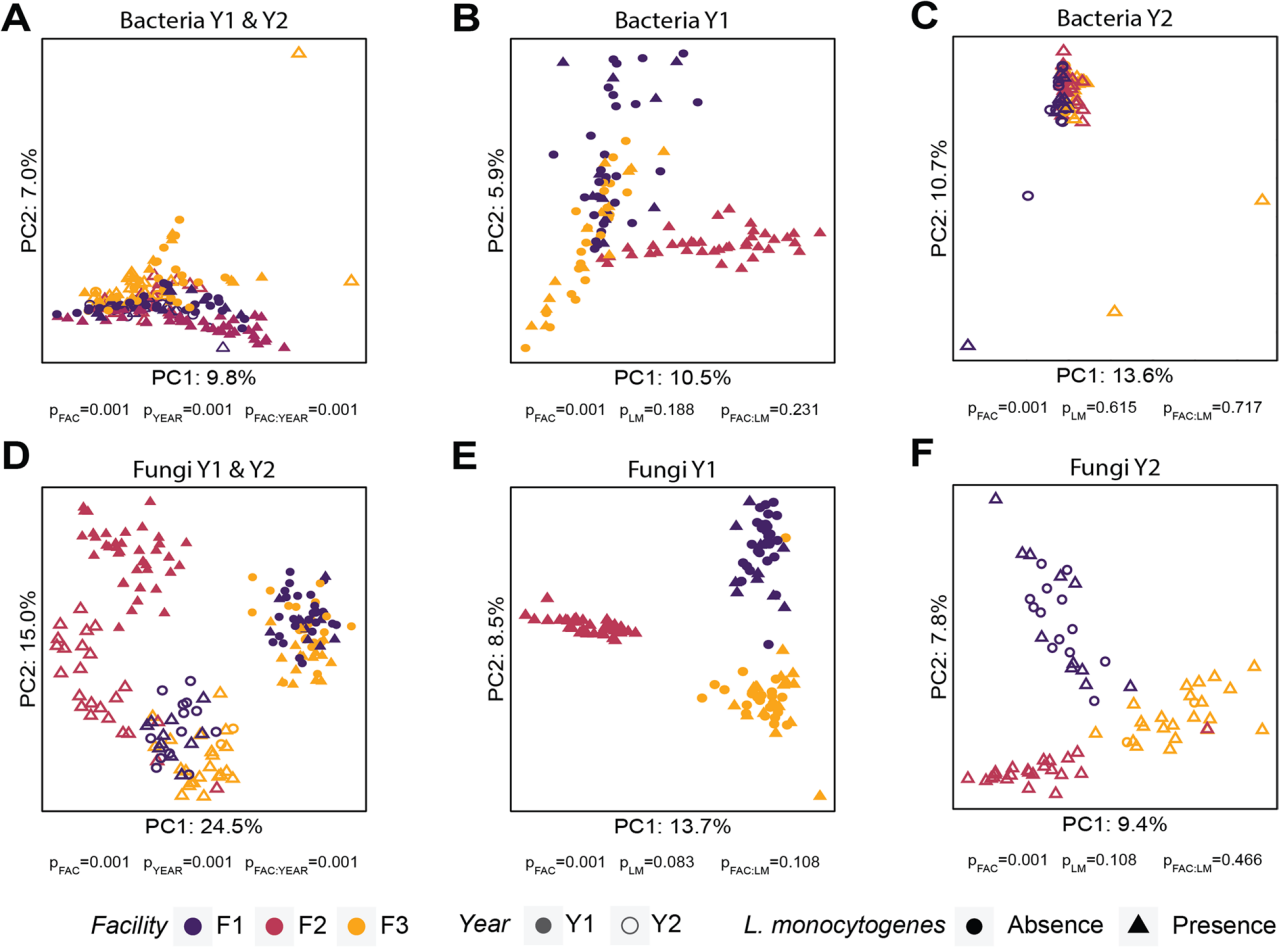
Seasonal changes in bacterial and fungal microbiota composition. Principal component plots show bacterial microbiota composition for samples collected in both years, (**A**) Y1 (**B**) and Y2 (**C**), and fungal microbiota for samples collected in both years, (**D**) Y1 (**E**) and Y2 (**F**). Each symbol in a plot represents an individual sample and the color of each sample symbol indicates the facility in which a sample was collected. The presence and absence of viable *L. monocytogenes*, as determined by an enrichment method, is shown in triangles and circles, respectively. Filled symbols denote samples collected in Y1 and unfilled symbols squares denote samples collected in Y2. The *p*-values in panels **A** and **D** were determined using a two-way PERMANOVA model that included the effect of facility (FAC), year (YEAR), and their interaction (FAC:YEAR). The *p*-values in panels **B**, **C**, **E**, and **F** from a two-way PERMANOVA model that included the effects of facility (FAC), presence of *L. monocytogenes* (LM), and their interaction (FAC:LM)

To assess changes in bacterial and fungal microbiota composition between the two packing seasons (Y1 and Y2), we analyzed amplicon sequencing data using a compositional analysis framework [51, 52]. Bacterial and fungal ASV tables were normalized using the CLR transformation, followed by beta-diversity calculation using the Aitchison distance. To determine if there was a significant difference in the microbiota composition of samples collected over two seasons, a two-way PERMANOVA model was used with facility, year, and their interaction effects. The results showed a significant interaction effect for both bacterial (*p* = 0.001) and fungal (*p* = 0.001) microbiota, indicating that the facility effect is dependent on the year of sampling (Fig. 2A, D). To further investigate whether the bacterial and fungal microbiota composition varied by facility, the bacterial and fungal ASV tables were split by year and analyzed independently. A one-way pairwise PERMANOVA was used to assess the effect of a facility on microbiota composition in samples collected in each year. In both seasons, the bacterial (*p* = 0.001) and fungal (*p* = 0.001) microbiota significantly differed among all compared facilities, suggesting spatial distinctiveness of microbiota (Fig. 2B,C,E, and F). Principal component analysis (PCA) was used to visualize clustering of samples based on bacterial and fungal microbiota composition. The first two principal components explained 16.8 and 39.5% of the variance in bacterial (Fig. 2A) and fungal (Fig. 2D) microbiota composition, respectively. The analysis of microbiota in samples collected in Y1 (Fig. 2B, E) showed that the first two principal components explained 16.4 and 22.2% of the variance in bacterial and fungal microbiota composition, respectively. For samples collected in Y2 (Fig. 2C, F), the top two principal components explained 24.3 and 17.2% of the variance in bacterial and fungal microbiota composition, respectively. Ordination of bacterial and fungal microbiota in samples collected in both seasons indicated a facility-specific clustering in which samples from individual facilities formed three distinct clusters (Fig. 2B, C, E, and F), which was consistent with the results of PERMANOVA analysis.

To visualize the taxonomic composition of bacterial and fungal microbiota samples collected from tree fruit packing facilities, we plotted the relative abundance of bacterial and fungal ASVs (Fig. S2). The three bacterial genera detected in highest relative abundance across the two seasons in F1 were *Flavobacterium* (13.5% in Y1 and 5.2% in Y2), *Acinetobacter* (8.2% in Y1 and 5.2% in Y2), and *Pseudomonas* (6.2% in Y1 and 4.7% in Y2). In F2, *Pseudomonas* (28.8%), *Flavobacterium* (14.1%), and *Stenotrophomonas* (5.7%) were the most abundant bacterial genera present in the facility during the first season, while in Y2 the three most abundant genera were *Pseudomonas* (4.8%), *Acinetobacter* (4.6%), and *Flavobacterium* (3.6%). In F3, the most abundant bacterial genera during the first sampling season were *Flavobacterium* (9.2%), *Chryseobacterium* (7.7%), and *Acinetobacter* (6.2%), while in Y2, *Acinetobacter* (5.1%), *Flavobacterium* (4.1%), and *Burkholderiaceae*_unclassified (3.1%) were the most abundant bacteria genera. Within the amplicon sequencing data for bacteria, only three samples collected in Y2, and none in Y1, had reads assigned to the genus *Listeria* with a relative abundance below 0.03%.

Analysis of fungal microbiota composition in Y1 revealed that the fungal genera detected in the highest relative abundance in F1 were *Ciliophora* (22.5%), *Aureobasidium* (17.6%), and Fungi_unclassified (10.8%) during Y1, while in Y2, the most abundant fungal genera were *Dipodascaceae*_unclassified (19.2%), *Yarrowia* (12.8%), and *Cutaneotrichosporon* (8.9%). In F2, the most abundant fungal genera throughout the two seasons were *Yarrowia* (45.0% in Y1 and 29.4% Y2), *Cutaneotrichosporon* (8.4% in Y1 and 7.5% in Y2), and *Dipodascaceae*_unclassified (8.2% in Y1 and 19.22% in Y2). In F3, the most abundant fungal genera during the first sampling season were *Cutaneotrichosporon* (25.7%), *Aureobasidium* (13.0%), and *Ciliophora* (10.9%), while in Y2, the most abundant fungal genera in F3 changed to *Cutaneotrichosporon* (25.4%), *Dipodascaceae*_unclassified (10.0%), and *Exophiala* (6.0%).

### Seven fungal ASVs were identified as members of the common and temporal core microbiota, and ninety and eighteen bacterial and fungal ASVs, respectively, were identified as network hubs

**Fig. 3:**
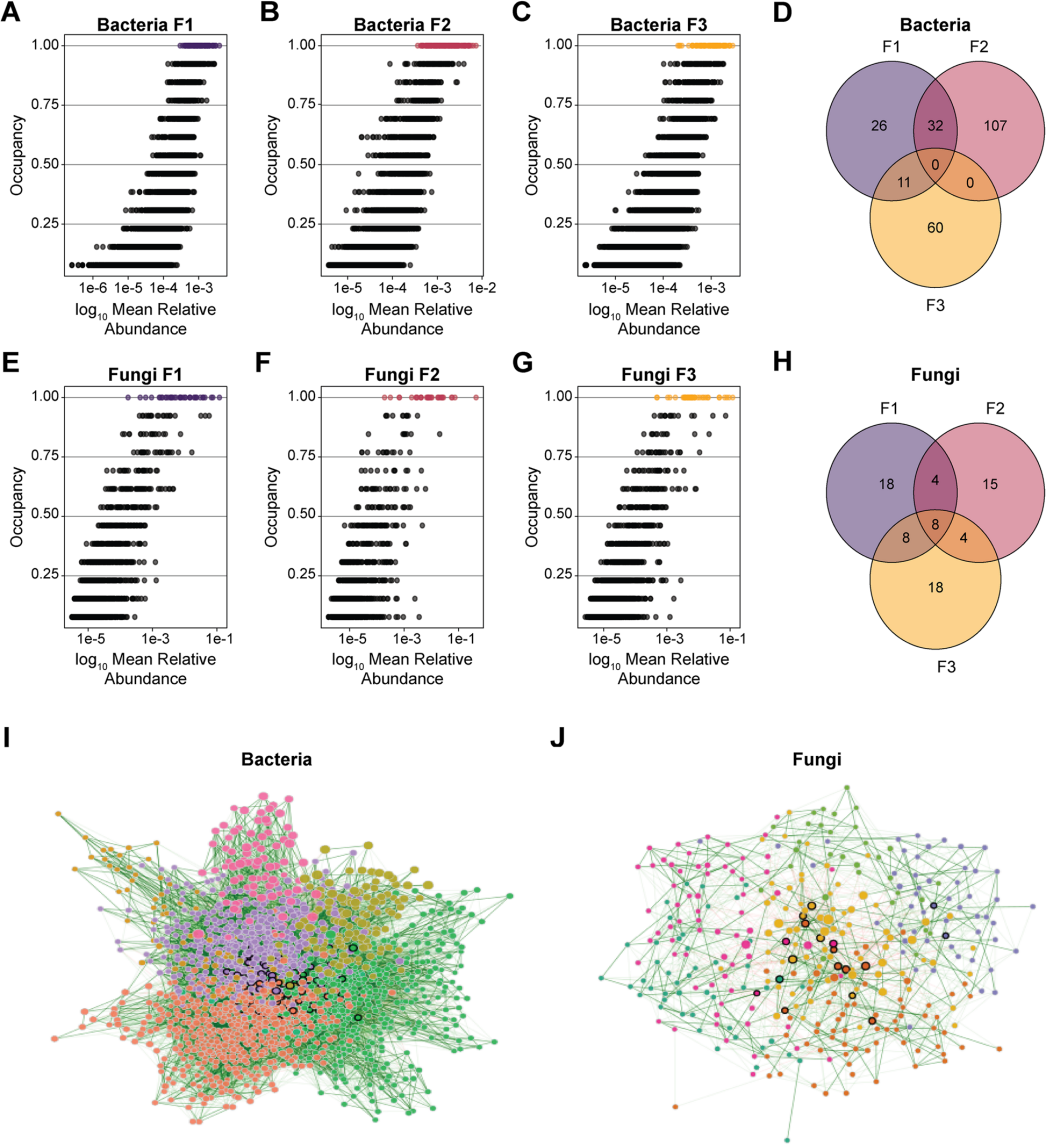
Abundance-occupancy distributions, overlapping core taxa, and co-occurrence networks. Abundance-occupancy distributions describing log_10_ mean relative abundance of individual ASVs and their occupancy for bacteria in F1 (**A**), F2 (**B**), and F3 (**C**), and for fungi in F1 (**E**), F2 (**F**), and F3 (**G**). Temporal core ASVs, defined as those present in all facilities at all sampling times throughout the 2 years of study (i.e., occupancy = 1), are shown in purple for F1, pink for F2, and yellow for F3. Venn diagrams show the number of core bacterial ASVs (**D**) and core fungal ASVs (**H**) shared between the three facilities. Networks show bacterial (**I**) and fungal (**J**) microbiota with an occupancy above 0.5 in any facility throughout the two seasons. Nodes represent ASVs and are color-coded by network cluster as determined by the fast greedy algorithm. Nodes marked with a black border line are network hubs, determined as those with the highest betweenness centrality. Edges in the network are color-coded to denote positive (green) and negative (red) associations. A higher transparency in the edge color indicates lower association value

Core microbiota (i.e., taxa that are consistently present in an environment) are hypothesized to play an important role in the ecology of microbial communities [64]. Different types of core microbiota have been described to date, including the common core (i.e., microbiota that is shared across spaces or hosts), temporal core (i.e., microbiota that is stable over time in a defined space), and ecological core (i.e., microbiota that shapes the organization of an ecological community) [36]. To identify common (i.e., shared across facilities) and temporal (i.e., shared across seasons) core ASVs present in the investigated tree fruit packing houses, we performed abundance-occupancy analysis that allowed us to detect ASVs that were present in at least one of the three samples collected from each facility throughout the two sampling years (i.e., taxa with occupancy equal to 1) [57, 65]. A total of 69, 139, and 71 bacterial ASVs were identified are part of the temporal core microbiota in F1, F2, and F3, respectively (Figs. 3A–C), none of which were common to all three facilities over two sampling seasons (Fig. 3D). A total of 38, 31, and 38 fungal ASVs were determined to be part of the temporal core microbiota in F1, F2, and F3, respectively (Figs. 3E–G), of which 8 ASVs where present in all three facilities throughout the two sampling seasons (Fig. 3H). The common temporal core fungal microbiota shared across facilities was comprised of ASVs belonging to 7 distinct taxonomic genera, including *Aureobasidium*, *Alternaria*, *Neocucurbitaria*, *Exophiala*, *Penicillium*, *Filobasidium*, and *Cystobasidium* (Table S4).

To assess whether the identified temporal core microbiota is likely of ecological importance in shaping the microbial communities within the studied environments, we employed a co-occurrence network analysis. Network analysis can be used to determine co-occurrence patterns in microbial communities and to identify hub taxa (i.e., taxa that are central for community stability). To reduce the sparsity of data, we included all ASVs that were detected in the tree fruit packing facilities in at least half of the samples collected throughout the 2 years (i.e., occupancy of 0.5) as previously described by Shade and Stopinsek [65]. To identify hub taxa (i.e., the most interconnected taxa in the network), we calculated the betweenness centrality parameter. Betweenness centrality measures the number of times an ASV is on the shortest path between other ASVs [66]. A high betweenness centrality between taxa suggests that they play an important role in maintaining the stability of a microbial community. Networks for bacterial microbiota (Fig. 3I) had 17,77 nodes (i.e., ASVs) connected within one network with 79.7% of the edges having a positive association. The bacterial co-occurrence network had a clustering coefficient of 0.097, a modularity of 0.331, and 7 unique clusters (i.e., network modules). Eighty-nine ASVs from 33 taxonomic genera were identified as network hubs with the highest betweenness centrality (Table S5). Networks for fungal microbiota (Fig. 3J) had 348 nodes (i.e., ASVs) connected in one network with 86.2% of the edges having a positive association. The fungal co-occurrence network had a clustering coefficient of 0.162, a modularity of 0.497, and 6 unique clusters. Eighteen ASVs from 14 taxonomic genera were identified as network hubs with the highest betweenness centrality (Table S5). Of the fungal hub taxa identified through network co-occurrence analyses, no ASVs were identified as part of both the shared and temporal core of the studied tree fruit packing facilities in this study.

### Twenty-three bacterial ASVs in F2, nineteen fungal ASVs in F1, eleven fungal ASVs in F2, and fifteen fungal ASVs in F3 contributed to observed differences in the total microbiota composition between seasons

**Fig. 4:**
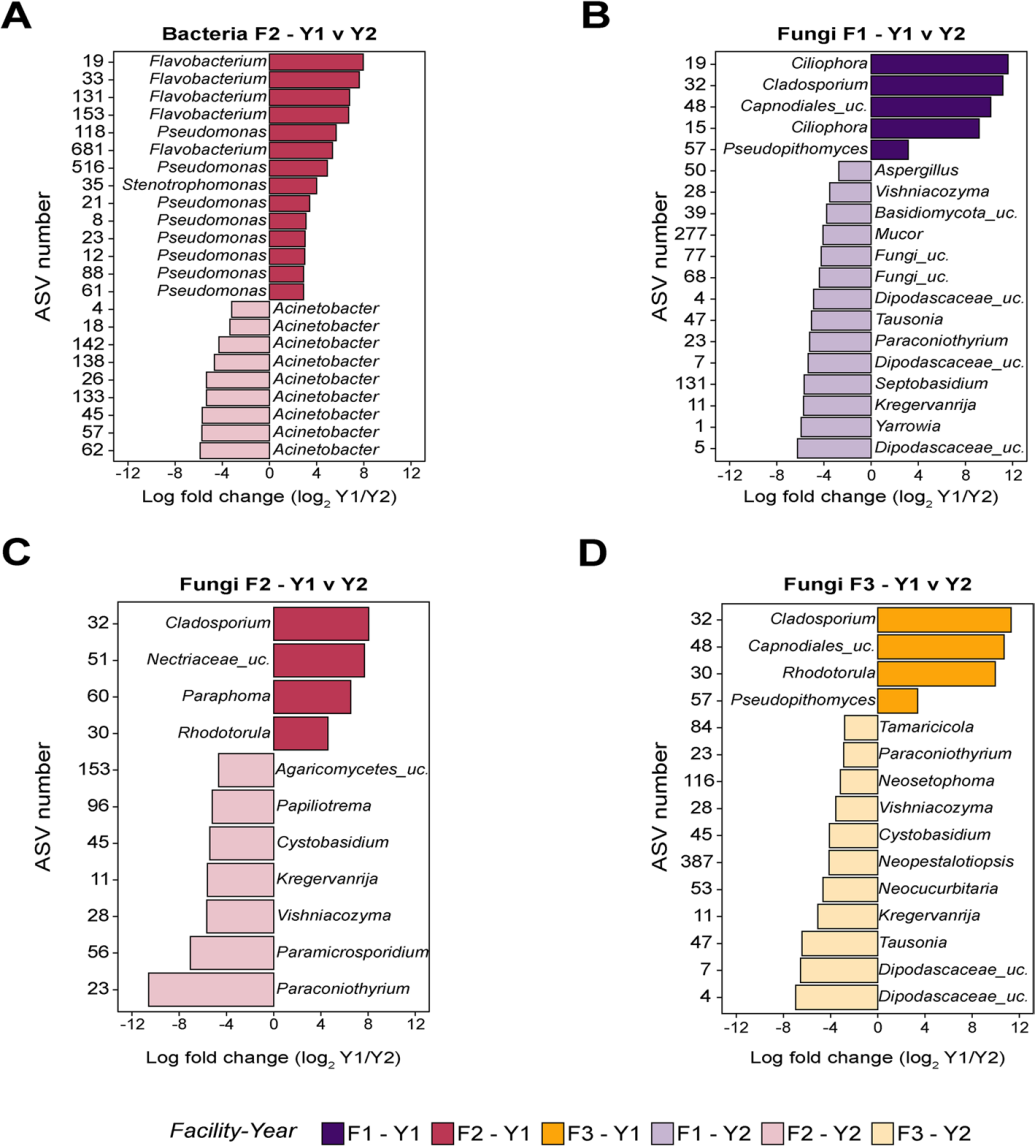
Differentially abundant bacterial and fungal ASVs between seasons. Bacterial ASVs identified as differentially abundant between season 1 (Y1) and 2 season (Y2) in facilities F2 (**A**), and fungal ASVs identified as differentially abundant between Y1 and Y2 in facilities F1 (**B**), F2 (**C**), and F3 (**D**). No differentially abundant bacterial ASV between seasons was found in F1 or F3. The ASVs shown in darker color were detected in significantly higher relative abundance in Y1, while ASVs shown in lighter color were detected as differentially abundant in Y2. Differences in relative abundance between seasons are shown as the logarithmic fold change of the mean relative abundance in Y1 to that of Y2. Negative values on the *x*-axis indicate that the relative abundance was higher in Y2

Given that the environmental microbiota was significantly different among three studied facilities, we further investigated whether the microbiota of each facility changed over two sampling seasons. Bacterial and fungal ASV tables were analyzed for each facility independently using a one-way PERMANOVA. The bacterial (*p* = 0.001) and fungal (*p* = 0.001) microbiota of all facilities (i.e., F1, F2, or F3) significantly differed between sampling years. To determine whether changes in relative abundance of ASVs contributed to the differences in the composition of bacterial and fungal microbiota composition between Y1 and Y2 within each facility, we conducted differential abundance analysis using ALDEx2 for each facility independently (Fig. 4). We detected 1169, and 0 differentially abundant bacterial ASVs in F1, F2, and F3, respectively, and 41, 34, and 30 differentially abundant fungal ASVs in F1, F2, and F3 between seasons, respectively. Of those, 23 bacterial ASVs in F2, 19 fungal ASVs in F1, 11 fungal ASVs in F2, and 15 fungal ASVs in F3 had an effect size above 1, suggesting a strong association [60]. In F1, five fungal ASVs (ASV19-*Ciliphora*, ASV32-*Cladosporium*, ASV48-*Caprodinales*_unclassified, ASV15-*Ciliophora*, and ASV57-*Pseudopithmyces*) had a significantly higher relative abundance in Y1 compared to Y2, and 14 fungal ASVs (ASV50-*Aspergillus*, ASV28-*Vishniacozyma*, ASV39-*Basidiiomycota*_unclassified, ASV277-*Mucor*, ASV77-Fungi_unclassified, ASV68-Fungi_unclassified, ASV4-*Dipodascaceae*_unclassified, ASV47-*Tausonia*, ASV23-*Paraconiothyrum*, ASV7-*Dipodascaceae*_unclassified, ASV131-*Septobasidium*, ASV11-*Kregervanrija*, ASV1-*Yarrowia*, and ASV5-*Dipodascaceae*_unclassified) had a significantly higher relative abundance in Y2 compared to Y1 (Fig. 4A). Of these, ASV28, ASV23, and ASV11 were also identified as network hubs (Table S6). In F2, 14 bacterial ASVs from the genera *Flavobacterium* (ASV19, ASV33, ASV131, ASV153, ASV68), *Pseudomonas* (ASV118, ASV516, ASV8, ASV23, ASV12, ASV88, ASV21, ASV61), and *Stenotrophomonas* (ASV35), and 4 fungal ASVs (ASV32-*Cladosporium*, ASV51-*Nectriaceae*_unclassified, ASV60-*Paraphoma*, and ASV30-*Rhodotorula*) were detected in a significantly higher relative abundance in Y1 when compared to Y2. Nine bacterial ASVs of the *Acinetobacter* genus (ASV4, ASV18, ASV142, ASV138, ASV26, ASV133, ASV45, ASV57, and ASV62) and 7 fungal ASVs (ASV153-A*garicomycetes*_unclassified, ASV96-*Papiliotrema*, ASV45-*Cystobasidium*, ASV11-*Kregervanrija*, ASV28-*Vishniacozyma*, ASV56-*Paramicrosporidium*, and ASV23-*Paraconiothyrium*) were detected in a significantly higher relative abundance in Y2 when compared to Y1 in the facility F2 (Fig. 4B, C). Of these, 4 fungal ASVs (ASV45, ASV11, ASV28, and ASV23) were identified as network hubs (Table S5). In F3, 4 fungal ASVs (ASV32-*Cladosporium*, ASV48-*Capnodiales*_unclassified, ASV30-*Rhodotorula*, and ASV57-*Pseudopithomyces*) had a significantly higher relative abundance in Y1 when compared to Y2, and 11 fungal ASVs (ASV84-*Tamaricola*, ASV23-*Paraconithyrium*, ASV116-*Neosetophoma*, ASV28-*Vishniacozyma*, ASV45-*Cystobasidium*, ASV387-*Neopestalotiopsis*, ASV53-*Neocucurbitaria*, ASV11-*Kregervanrija*, ASV47-*Tausonia*], ASV7-*Dipodascaceae*_unclassified, and ASV4-*Dipodascaceae*_unclassified) had a higher relative abundance in Y2 compared to Y1. Of these, 5 fungal ASVs (ASV23, ASV116, ASV28, ASV45, and ASV11) were identified as network hubs (Table S5).

### Total microbiota composition was not associated with *L. monocytogenes* presence; however, a subset of ASVs was indicative of its presence

**Fig. 5:**
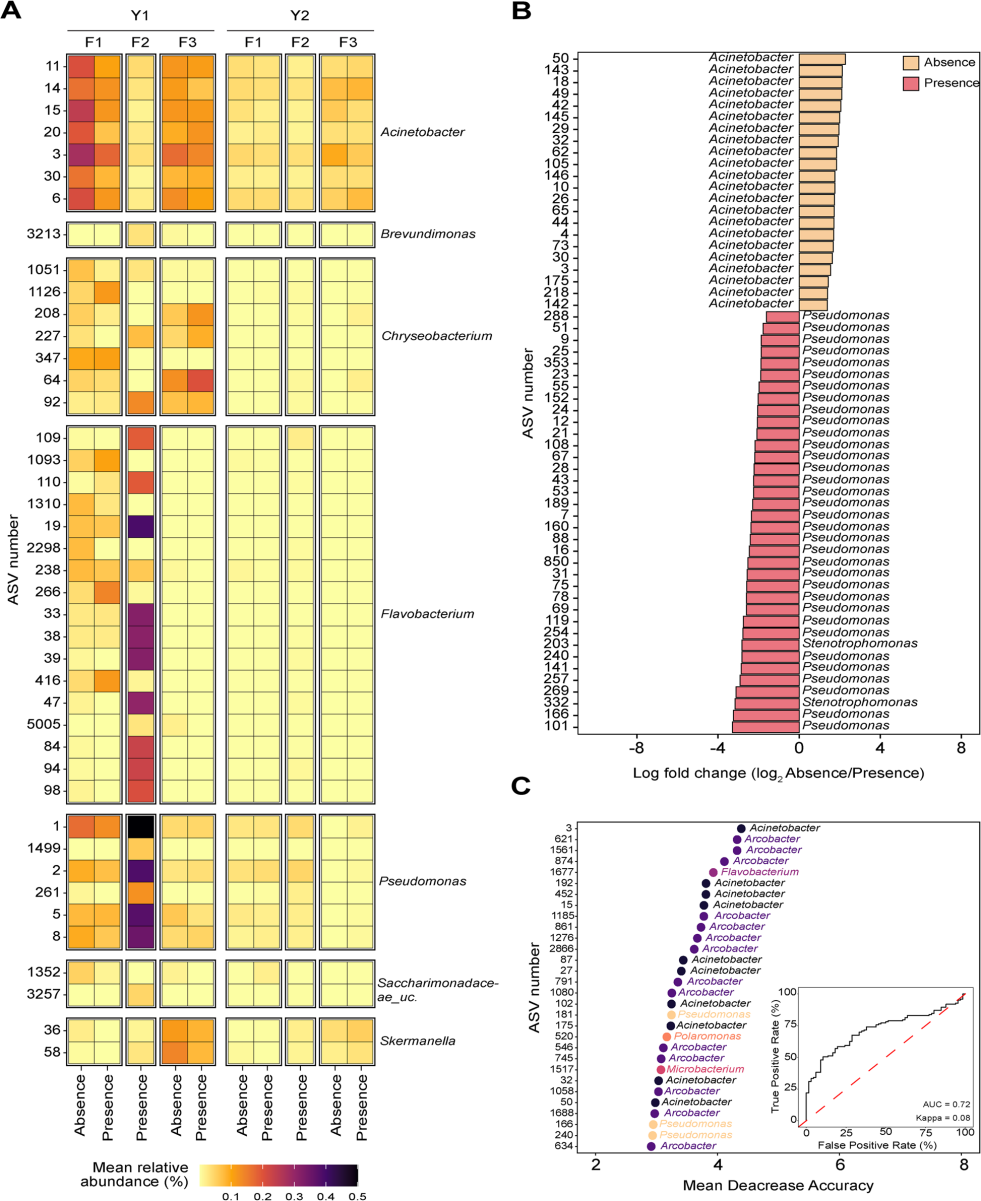
Differences in bacterial microbiota between *L. monocytogenes*-positive and -negative samples. A heatmap shows the difference in mean relative abundance of bacterial ASVs in *L. monocytogenes*-positive and -negative samples (**A**) for all ASVs that had a relative abundance above 1% in at least one sample across the two seasons. Significantly differentially abundant bacterial ASVs identified in samples in year 1 (**B**). In year 2, there were no significant differentially abundant ASVs identified between *L. monocytogenes*-positive and -negative samples. Differences in the relative abundance between *L. monocytogenes*-positive and -negative samples are shown as the log fold change of the mean relative abundance in *L. monocytogenes*-negative samples to that in *L. monocytogenes*-positive samples. ASVs shown in orange were detected in a significantly higher relative abundance in *L. monocytogenes-*negative samples and ASVs shown in pink were detected in a significantly higher relative abundance in *L. monocytogenes-*positive samples. The top 30 bacterial ASVs identified by a random forest model as most informative for classification of samples into *L. monocytogenes*-positive and *L.* monocytogenes-negative categories are shown in the panel (**C**). These ASVs had the highest mean decrease in the model accuracy (mtry = 153, ntree = 1500, accuracy = 65.1%). The inserted plot shows the area under the curve (AUC) and kappa values for the random forest model. “Uc” indicates ASVs that were not classified at the genus level

**Fig. 6:**
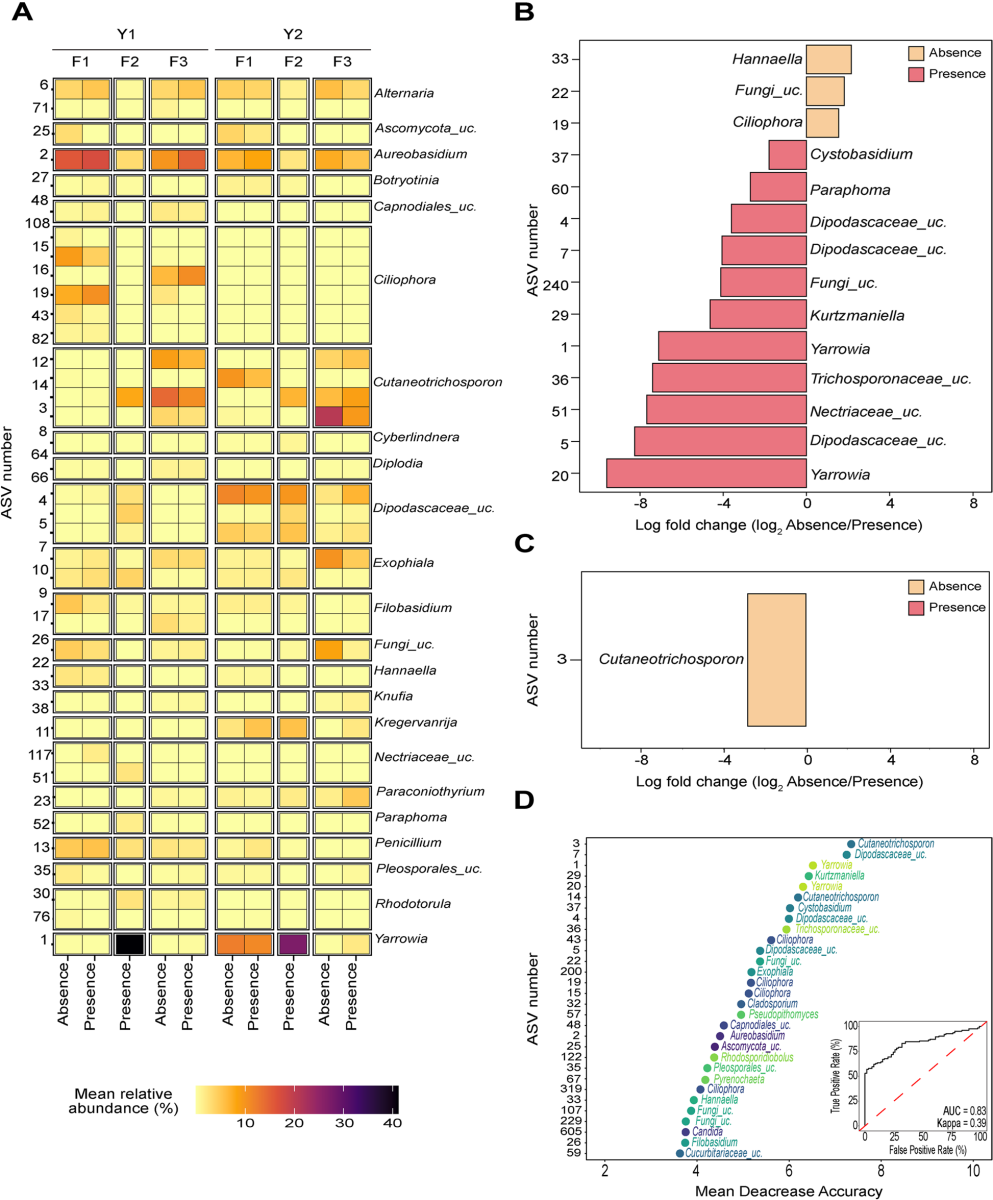
Differences in fungal microbiota between *L. monocytogenes***-**positive and -negative samples. A heatmap shows the difference in mean relative abundance of fungal ASVs in *L. monocytogenes*-positive and -negative samples for all ASVs that had a relative abundance above 10% in at least one sample across the two seasons (**A**). Significantly differentially abundant fungal ASVs identified in samples in in year 1 and in year 2 are shown in panels **B** and **C**, respectively. Differences in the relative abundance between *L. monocytogenes*-positive and -negative samples are shown as the log fold change of the mean relative abundance in *L. monocytogenes*-negative samples to that in *L. monocytogenes*-positive samples. ASVs shown in orange were detected in significantly higher relative abundance in *L. monocytogenes-*negative samples and ASVs shown in pink were detected in a significantly higher relative abundance in *L. monocytogenes-*positive samples. The top 30 fungal ASVs identified by a random forest model as most informative for classification of samples into *L. monocytogenes*-positive and *L.* monocytogenes-negative categories are shown in the panel **D**. These identified ASVs had the highest mean decrease in model accuracy (mtry = 26, ntree = 1000, accuracy = 74.9%). The inserted plot shows the area under the curve (AUC) and kappa values for the random forest model. “Uc” indicates ASVs that were not classified at the genus level

We investigated whether the presence of *L. monocytogenes* was associated with the composition of bacterial and fungal microbiota. To assess this, a two-way PERMANOVA was applied to the Aitchison distance matrix with a model that included the effects of the facility, *L. monocytogenes* presence, and their interaction. A significant effect of facility (*p* = 0.001) on the bacterial and fungal microbiota composition was detected in both seasons (Fig. 2B, C, E, and F). However, the effect of viable *L. monocytogenes* presence on both bacterial (*p*_Y1_ = 0.188; *p*_Y2_ = 0.717) and fungal microbiota (*p*_Y1_ = 0.083; *p*_Y2_ = 0.108) during both seasons was not significant (Fig. 2B,C,E, and F). We further investigated whether individual bacterial or fungal ASVs were present in higher relative abundance between *L. monocytogenes*-positive and negative samples. By examining relative abundances of the most abundant ASVs (those present in at least 1% relative abundance in any sample over 2 years for bacteria, and those present over 10% relative abundance for fungi), we found that some ASVs had a higher relative abundance in samples that were positive for viable *L. monocytogenes*. Specifically, bacterial genera *Chryseobacterium* (ASV208, ASV227, ASV64, and ASV92), *Flavobacterium* (ASV109, ASV110, ASV19, ASV33, ASV38, ASV39, ASV47, ASV84, ASV94, and ASV98), *Pseudomonas* (ASV1, ASV2, ASV5, and ASV8) (Fig. 5A), and fungal genera *Aureobasidium* (ASV2), *Ciliophora* (ASV00016 and ASV19), *Kregervanrija* (ASV11), and *Yarrowia* (ASV1) (Fig. 6A), had, on average, a consistently higher relative abundance in *L. monocytogenes*-positive samples, compared to *L. monocytogenes*-negative samples. To statistically test whether these taxa are significantly more or less abundant when samples are *L. monocytogenes*-positive, we carried out a differential abundance analysis using ALDEx2, for the 2 years combined, as well as independently for each year. When analyzing the 2-year dataset, no bacterial ASV was significantly differentially abundant either on *L. monocytogenes*-positive or -negative samples. Analysis of samples collected in Y1 identified 58 bacterial ASVs as differentially abundant (Fig. 5C). Thirty four ASVs from the *Pseudomonas* genus (ASV288, ASV51, ASV9, ASV25, ASV323, ASV23, ASV55, ASV152, ASV24, ASV12, ASV21, ASV108, ASV67, ASV28, ASV43, ASV53, ASV189, ASV7, ASV160, ASV88, ASV16, ASV850, ASV31, ASV75, ASV78, ASV69, ASV119, ASV254, ASV240, ASV141, ASV257, ASV269, ASV166, and ASV101) and two ASVs from the *Stenotrophomonas* genus (ASV203 and ASV332) were detected in a significantly higher relative abundance in samples that tested positive for *L. monocytogenes* compared to *L. monocytogenes*-negative samples. Twenty-two ASVs from the *Acinetobacter* genus (ASV50, ASV143, ASV18, ASV49, ASV42, ASV145, ASV29, ASV32, ASV62, ASV105, ASV146, ASV10, ASV26, ASV65, ASV44, ASV4, ASV73, ASV30, ASV3, ASV175, ASV218, and ASV142) were detected in a significantly higher relative abundance in samples that tested negative for *L. monocytogenes*. Analysis of Y2 samples did not yield any differentially abundant bacterial ASVs between *L. monocytogenes*-positive and *L. monocytogenes*-negative samples.

When analyzing the 2-year dataset together, a total of 16 fungal ASVs were identified as significantly differentially abundant between *L. monocytogenes*-positive samples and *L. monocytogenes*-negative samples. Three ASVs from *Dipodascaceae* (ASV4, ASV7, and ASV5), two ASVs from *Yarrowia* (ASV1 and ASV20), one ASV from *Cystobasidium* (ASV37), one ASV from *Cutaneotrichosporon* (ASV3), one ASV from *Cucurbitariaceae*_unclassified (ASV59), one ASV from *Kurtzmaniella* (ASV29), and one ASV from *Trichosporonaceae_*unclassified (ASV36) were detected in a significantly higher relative abundance in samples that tested positive for *L. monocytogenes*. ASV33 [*Hannaella*], ASV57 [*Pseudopithomyces*], ASV22 [Fungi_unclassified], ASV32 [*Cladosporium*], ASV67 [*Pyrenochaeta*], and ASV2 [*Aureobasidium]* were detected in a significantly higher relative abundance in samples that tested negative for *L. monocytogenes*. Analysis of Y1 samples independently revealed a total of 14 fungal ASVs as significantly differentially abundant between *L. monocytogenes*-positive samples and *L. monocytogenes*-negative samples. Two ASVs from *Yarrowia* genus (ASV1 and ASV20), three ASVs from *Dipodascaceae*_unclassified (ASV4, ASV7, and ASV5), ASV37-*Cystobasidium*, ASV60-*Paraphoma*, ASV240-Fungi_unclassified, ASV29-*Kurtzmanella*, and ASV36-*Trichosporonaceae*_unclassified were detected in a significantly higher relative abundance in samples that tested positive for *L. monocytogenes* using the enrichment detection method (Fig. 6B). In contrast, ASV33-*Hannaella*, ASV22-Fungi_unclassified, and ASV19-*Ciliophora* were detected in a higher relative abundance in samples that tested negative for *L. monocytogenes* (Fig. 6B). Analysis of microbiota of Y2 samples independently revealed 1 differentially abundant ASV (ASV3 – *Cutaneotrichosporon*), which was detected in a significantly higher relative abundance in *L. monocytogenes*-negative samples (Fig. 6C).

Since the differences in bacterial and fungal microbiota composition may be driven by F2 data, in which all collected samples tested positive for *L. monocytogenes* (Fig. 1B), the differential abundance analysis was repeated for each year independently, excluding F2. These analyses resulted in no bacterial or fungal ASVs identified as significantly differentially abundant in Y1 or Y2, further confirming that the significance of identified differentially abundant taxa was likely driven by microbiota composition of samples from F2. Further, all ASVs detected as differentially abundant, with the exception of fungal ASV3, had an effect size smaller than one, suggesting a weak association.

To further evaluate whether a subset of ASVs is indicative of the presence of *L. monocytogenes* in the monitored tree fruit packing facilities, random forest was applied to classify samples into *L. monocytogenes*-positive and -negative categories based on the microbiota composition. Bacterial and fungal ASV tables were used as predictor variables for the classification, and the presence or absence of *L. monocytogenes* in each sample was used as an outcome. For bacterial ASVs, the random forest model with the highest accuracy of classification (mtry = 153, ntree = 1500, accuracy = 65.1%, AUC = 0.72, kappa = 0.08) identified ASV3 [*Arcobacter*], ASV 621 [*Arcobacter*], and ASV1561 [*Pseudomonas*] as ASVs with the highest contribution to classification accuracy (Fig. 5C). Of the thirty bacterial taxa that were most informative for classification, ASVs from the genus *Microbacterium* (ASV1517) and *Pseudomonas* (ASV166, ASV181, and ASV240) had an increased mean relative abundance in *L. monocytogenes-*positive samples. For fungal ASVs, the random forest model with the highest accuracy of classification (mtry = 26, ntree = 1000, accuracy = 74.9%, AUC = 0. 83, kappa = 0.39) identified ASV3 [*Cladosporium*], ASV7 [*Candida*], and ASV1 [*Yarrowia*], as the ASVs that were most informative for classification (Fig. 6D). Of the thirty most informative fungal taxa, ASVs from the genera Yarrowia (ASV1, ASV20), *Kurtzmaniella* (ASV29), *Cutaneotrichosporon* (ASV3), *Trichosporonaceae*_unclassified (ASV36), *Cystobasidium* (ASV37), *Cucurbitariaceae*_unclassified (ASV59), and *Dipodascaceae*_unclassified (ASV4, ASV5, ASV7) had an increased mean relative abundance in *L. monocytogenes*-positive samples. Six bacterial ASVs (ASV50, ASV32, ASV3, ASV, 175, ASV240, and ASV166) and 16 fungal ASVs (ASV33, ASV19, ASV57, ASV22, ASV32, ASV2, ASV37, ASV3, ASV4, ASV7, ASV1, ASV59, ASV29, ASV5, ASV20, and ASV36) identified by random forest analysis as variables of importance for classification (Figs. 5D and 6D) were also found to be differentially abundant between *L. monocytogenes*-positive and -negative samples (Figs. 5B,C and 6B,C). As in the differential abundance analysis, the random forest analysis was likely strongly influenced by samples from F2 where all samples were positive for *L. monocytogenes*.

### *L. monocytogenes* reads were detected by direct sequencing of three environmental microbiome samples with Nanopore MinION

**Fig. 7:**
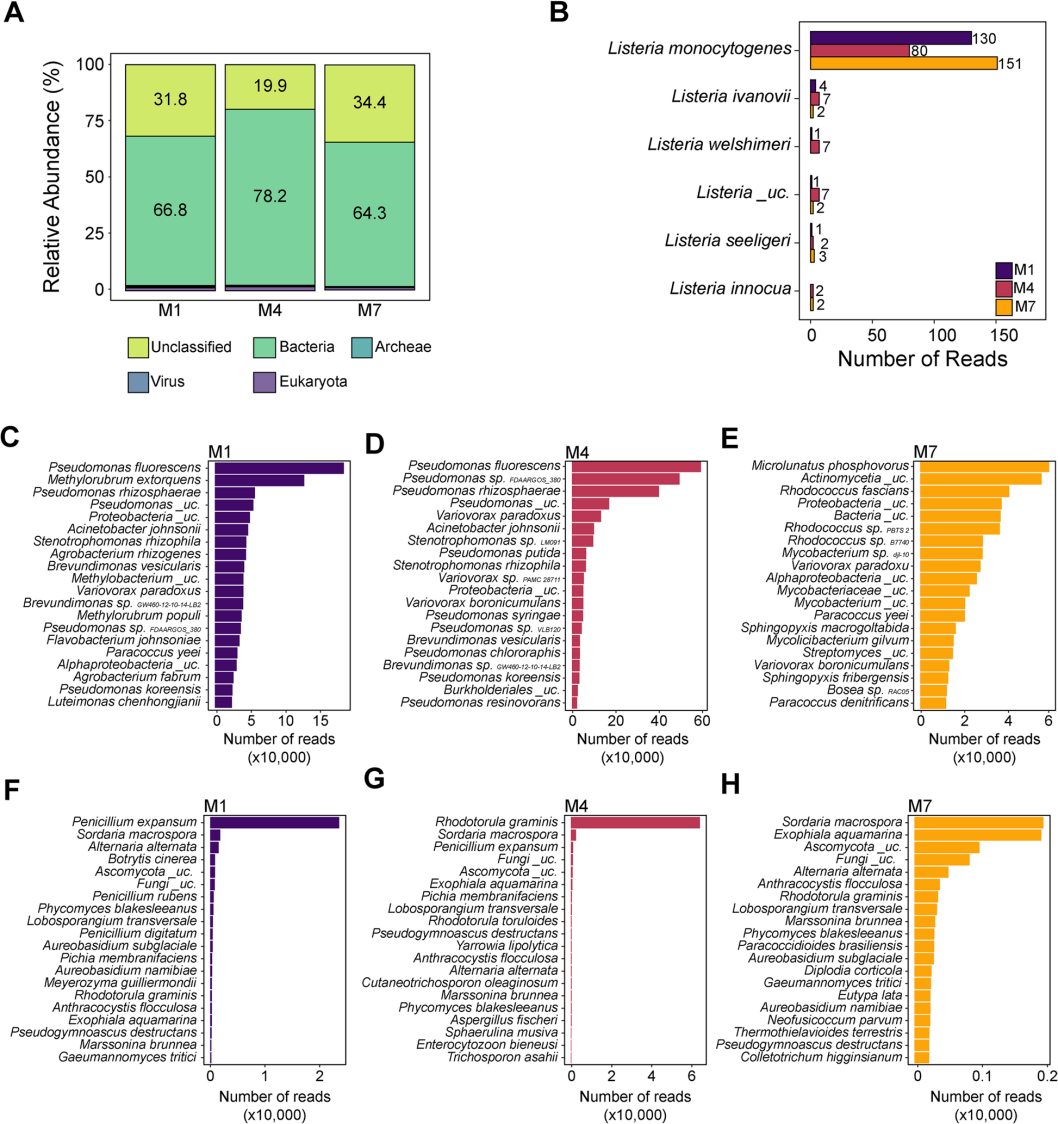
Comparison of the taxonomic composition of three samples determined by Illumina amplicon sequencing and Nanopore shotgun sequencing. The kingdom level classification of Nanopore reads for three environmental samples (M1, M4, and M7) collected from three tree fruit packing houses is shown in the panel (**A**). *Listeria* spp. diversity as identified by Nanopore sequencing is shown in the panel (**B**). The top 20 most abundant bacterial species present in M1 (**C**), M4 (**D**), and M7 (**E**), and the top 20 most abundant fungal species present in M1 (**F**), M4 (**G**), and M7 (**H**), as determined by shotgun metagenomic sequencing using Nanopore long-read technology are also shown. “Uc” stands for “unclassified”

To evaluate the feasibility of using Nanopore long-read metagenomic sequencing to detect *L. monocytogenes* DNA directly from metagenomic DNA extracted from environmental samples, we carried out a proof-of-concept experiment using three *L. monocytogenes-*positive environmental samples. All selected samples were collected on the same date from the washing section of F1 (sample M1 = 1s022119), F2 (sample M4 = 4s022119), and F3 (sample M7 = 7s022119) and had tested positive for *L. monocytogenes* using the FDA BAM enrichment method (Table S1). Each sample was sequenced on a separate flow cell, which resulted in a total of 4,042,609, 5,223,341, and 3,364,310, reads with a median read length of 3257, 4115, and 4405 bases for M1, M4, and M7, respectively. Of the total reads obtained for the samples, 64.3–78.2% of the reads were classified as bacteria, 0.02–0.07% of the reads were classified as archaea, 0.02–0.1% of the reads were classified as virus, and 0.98–1.75% of the reads were classified as eukaryote (Fig. 7A). Of the eukaryote reads, 0.8–1.6% of the reads were classified as fungi. Sequences that remained unclassified may belong to organisms not included in the classifier algorithm (e.g., plant and animal DNA), most likely DNA coming from fruit leaves and debris left in the environment, or from unspecific reads. A total of 137 (0.005% of bacterial reads), 105 (0.002% of bacterial reads), and 160 (0.007% of bacterial reads) reads were assigned to the genus *Listeria* in samples M1, M4, and M7, respectively (Fig. 7B). *L. monocytogenes* was the main species identified within *Listeria* genus in all three samples. Specifically, a total of 130 (0.004% of bacterial reads), 80 (0.001% of bacterial reads), and 151 (0.006% of bacterial reads) *L. monocytogenes* reads were detected in M1, M4, and M7, respectively (Fig. 7B). Further, other *Listeria* spp. were detected in all samples using Nanopore sequencing, including *Listeria ivanovii*, *Listeria welshimeri*, *Listeria seeligeri*, and *Listeria innocua* (Fig. 7B).

Given the likely differences in the sequencing depth between amplicon and shotgun sequencing, a direct comparison of the microbiota composition between the two sequencing approaches is challenging. In contrast to Illumina amplicon sequencing, Nanopore shotgun sequencing allowed for a taxonomic classification of reads at the species level. Thus, we examined the most prevalent bacterial and fungal species present in each sample. In M1, the three most abundant bacterial species were *Pseudomonas fluorescens*, *Methylorubrum extorquens*, and *Pseudomonas rhizosphaerae* (Fig. 7C), while the three most abundant fungal species were *Penicillium expansum*, *Sordaria macrospora*, and *Alternaria alternata* (Fig. 7F). In M4, the three most abundant bacterial species were *Pseudomonas fluorescens*, *Pseudomonas* sp. FDAARGOS_380, and *Pseudomonas rhizosphaerae* (Fig. 7D), while the three most abundant fungal species were *Rhodotorula graminis*, *Sordaria macrospora*, and *Penicillium expansum* (Fig. 7G). In M7, the three most abundant bacterial species were *Microlunatus phosphovorus*, *Actinomycetia_*unclassified, and *Rhodococcus fascians* (Fig. 7E), while the three most abundant fungal species were *Sordaria macrospora*, *Exophiala aquamarine*, and *Ascomycota*_unclassified (Fig. 7H).

## Discussion

### *L. monocytogenes* occurrence was high in all three monitored tree fruit packing facilities, and it significantly increased from first to second season in facility F3

Throughout the two seasons, we detected *L. monocytogenes* in a high proportion of samples collected from floor under tree fruit processing lines in tree monitored packing facilities. Further, we observed that the proportion of samples that tested positive for *L. monocytogenes* in F1 and F3 increased from year 1 to year 2, particularly in the waxing area. Waxing is a practice commonly used in the tree fruit packing industry to give fruit a glossy appearance that is appreciated by consumers and that extends the fruit shelf life [67]. Similarly, in the previous study within these three packing facilities, we identified the waxing area as a high *L. monocytogenes* occurrence spot [6]. Furthermore, a recent study on the prevalence of *Listeria* species within apple packing facilities in Washington state identified the waxing unit operation as the area where *Listeria* was most frequently isolated [68]. The application of shellac-based wax coating on apples had a strong impact on the fungal and bacterial diversity and community composition of apple fruit [69] and has been found to significantly facilitate the long-term survival of *L. monocytogenes* [67]. Due to the hydrophobic nature of wax, its residues are difficult to remove from the tree fruit packing equipment and environment (e.g., bristles of brushes, floor) during cleaning and sanitizing operations. We therefore hypothesize that wax residues could create a favorable microenvironment for *L. monocytogenes* by creating a protective coating that reduces the diffusion of sanitizers, which could explain the repeated isolation of *L. monocytogenes* from the waxing area. However, further research is needed to determine how residual wax in the environment affects the survival and persistence of *L. monocytogenes* and to evaluate best approaches for wax removal*.* Overall, given the high occurrence of *L. monocytogenes* in the studied tree fruit packing facilities, an in-depth risk assessment is needed to identify the source of contamination and whether the detected strains are similar to those isolated from human illness cases.

### The composition of environmental microbiota differed significantly among monitored facilities and between seasons

We detected a seasonal shift in bacterial and fungal microbiota composition in monitored facilities over two seasons. Specifically, the significantly different composition of bacterial and fungal microbiota observed among three monitored facilities in Y1 changed in Y2, although it remained significantly different among facilities. This suggests that the composition of microbiota in these environments is facility-specific and varies over seasons, due to factors that were not assessed in this study. The seasonal differences in the bacterial microbiota composition within F1 and F3 were not due to changes in relative abundance of specific ASV, suggesting that the differences were due to introduction of new microbial species or loss of previously present species. Further, the seasonal differences observed in the bacterial microbiota of F2, and in the fungal microbiota of each facility were partially due to some high abundant taxa, as determined by differential abundance analysis (Fig. 4). The microbiota of the built environments can vary based on the building design, geographic location, ventilation, the outdoors, and its human occupants [9]. The samples in this study were collected from the wet processing area to which fruit is brought directly from the orchard. Hence, the variation in microbiota composition among facilities and between harvesting seasons may be due to changes in the microbiota composition in the orchard soil, which is introduced into facilities with fruit and harvesting bins. Some of the bacterial and fungal taxa detected in a significantly higher relative abundance between seasons are often associated with soil or plants (e.g., *Pseudomonas* [70], *Stenotrophomonas* [71, 72], *Flavobacterium* [73], *Acinetobacter* [74], *Aspergillus* [75],* Cladosporium* [76], *Mucor* [77], *Vishniacozyma* [78]), suggesting that seasonal changes in the microbiota of the orchard environment that is introduced with the fruit may influence the microbiota of fruit packing-built environments. Indeed, a recent study has shown that the microbiota composition of soil collected from apple orchards varied over the course of the year, exhibiting seasonal variability [79]. Further, studies of apple microbiota identified *Aureobasidium*, *Cladosporium*, and *Vishniacozyma* as highly abundant taxa on apple skins [80], and on Royal Gala apples [81]. However, we did not collect soil or apple samples in this study to test whether and to what extent the microbiota found in food processing environmental samples is influenced by soil and apple microbiomes. Further studies could employ source-tracking methods to assess the origin of the microbiota found in tree fruit packing facilities.

Some bacterial and fungal genera that were identified as differentially abundant between sampling seasons have previously been detected in water and marine environments (e.g., *Pseudomonas* [82], *Flavobacterium* [83], *Acinetobacter* [74], *Aspergillus* [75], *Cladosporium* [76]). This suggests that variability in the microbiota of water used within the facilities may potentially affect the microbiota in the food processing environment. An additional factor that may affect the composition of microbiota in the built environment is the presence of personnel working in the facilities and the rigor of the cleaning and sanitizing procedures practiced in facilities. Changes in personnel between harvesting seasons are common in these types of food operations due to seasonal nature of work and failure to provide effective and accessible food safety training is likely to result in poor hygienic and sanitation practices. The increased presence of bacteria that are normally associated with human skin (e.g., *Acinetobacter* [74]) during the second season of sampling provides some evidence of the influences of the human presence on the microbiota of fruit packing facilities. Further studies that employ source-tracking methods could aid in determining whether the causes of spatial and temporal variation of microbiota composition in these food processing environments could be attributed primarily to soil, water, human activity, or other factors such as cleaning and sanitizing protocols.

Modification of the cleaning and sanitation standard operating procedures, such as changes in the chemicals used for cleaning and sanitizing could influence the microbiota composition. Before the start of the sampling in each season, each facility’s manager was asked to provide information on the cleaning and sanitizing procedures and chemicals used in their facility. Throughout this study, changes in sanitizing chemicals used between the two sampling seasons were reported only in facility F1. Despite the changes in the sanitizer product applied in F1, both sanitizers were based on quaternary ammonium chemistry. F2 and F3 reported using the same, peroxyacetic acid-based sanitizer, throughout the two seasons.

To date, very few studies were conducted to determine longitudinal changes in microbiota composition in food processing environments [19, 34]. Similar to our results, fermentation-based food manufacturing facilities (i.e., wine, cheese production) showed temporal and spatial variation of the microbiota between different areas in a facility [19, 34]. Nonetheless, the production of fermented products relies on the introduction of starter cultures that can shape the environmental microbiome composition, which is not the case in tree fruit packing facilities. The detection of seasonal changes in the composition of bacterial and fungal microbiota in food processing and packing facilities, including this study, demonstrates that cross-sectional characterization of microbiota in food processing facilities may not be representative of microbiota observed in facilities over time. Hence, longitudinal and spatial characterization of food processing-built environment microbiota is needed to identify core and accessory microbiota for further assessment of their role in food quality and safety.

### ASVs from bacterial genera *Pseudomonas*, *Acinetobacter, Flavobacterium, Stenotrophomonas*, and *Chryseobacterium*, and from fungal genera *Yarrowia, Aureobasidium, Ciliophora, Cutaneotrichosporon, Dipodascaceae*_unclassified, and* Exophiala* were detected in highest relative abundance in three monitored facilities over two seasons

ASVs from bacterial genera *Pseudomonas*, *Acinetobacter*, *Flavobacterium*,* Stenotrophomonas*, and *Chryseobacterium*, and from fungal genera *Yarrowia*, *Aureobasidium*, *Ciliophora*, *Cutaneotrichosporon*, *Dipodascaceae*_unclassified, and *Exophiala* were consistently present in a high relative abundance in the surveyed tree fruit packing facilities*.* Similar to our finding, previous studies have found *Pseudomonas* [10, 19, 28, 29, 31], *Flavobacterium* [19], *Acinetobacter* [28, 29, 31], *Stenotrophomonas* [10], and *Aureobasidium* [19] in high relative abundances within the microbiota of plant-based food processing facilities. These bacterial and fungal genera are typically found also in soils, in contact with plants (e.g., *Pseudomonas* [70], *Stenotrophomonas* [71, 72], *Flavobacterium* [73], *Acinetobacter* [74], *Chryseobacterium* [84], *Dipodascaceae* [85], *Aureobasidium* [86–88], *Cutaneotrichosporon* [89, 90]), in water (e.g., *Pseudomonas* [82], *Flavobacterium* [83], *Acinetobacter* [74], *Chryseobacterium* [91],* Yarrowia* [92], *Exophiala* [93]), on insects (e.g., *Yarrowia*), on human skin (e.g., *Cutaneotrichosporon* [89], *Acinetobacter* [74], *Exophiala* [93]), or on apple skins (e.g., *Aureobasidium* [80, 81]). Hence, their recurrent presence in tree fruit packing facilities may be due to the recurring introduction of produce from the orchard. Additionally, some members of these taxonomic genera are psychrotrophs, which allows them to survive and grow at lower temperatures found in the packing facilities. The investigated tree fruit packing environments had a mean temperature of 15.0 ± 3.6 °C throughout the study, which is an ideal temperature for psychrotrophs, including *L. monocytogenes*, to outcompete other organisms and thrive in. Furthermore, members of these bacterial families (e.g., *Pseudomonas* [82], *Flavobacterium* [73]) as well as yeasts and other dimorphic fungi, such as *Aureobasidium* and *Exophiala* [94], can strongly attach to surfaces or form biofilms, allowing them to persist within the packing facility environment. Some species of *Dipodascaceae* are known to reside on unclean food processing equipment and are commonly referred to as “machinery mold” [95, 96]. Given that all the samples were collected during operating hours, we cannot draw conclusions as to whether these taxa have persisted in the environment through cleaning and sanitizing. However, the ability of these taxa to form strong biofilms and thrive at lower temperature suggests that they might be challenging to control. Further research is needed to determine the composition of microbiota that persists in fruit packing environments through cleaning and sanitizing as those taxa are more likely to affect *L. monocytogenes* survival and persistence over time.

### ASVs from fungal genera *Aureobasidium, Alternaria, Neocucurbitaria, Exophiala, Penicillium, Filobasidium*, and *Cystobasidium* were identified as temporal common core microbiota in monitored fruit packing facilities

Some microorganisms introduced to food processing facilities are transient, meaning that they are removed with routine cleaning and sanitizing, whereas others may colonize the environment and persist over time. Persistent taxa that are detected over an extended period of time (i.e., temporal core microbiota) are hypothesized to play an important role in the microbial ecology of food processing environments. In this study, we aimed to identify the common, temporal, and ecological core microbiota [36] of the monitored tree fruit packing facilities. While we detected temporal core bacterial ASVs within each facility, no bacterial ASV was found to be common temporal core in all three tree fruit packing facilities over two sampling seasons. In contrast, we detected temporal common core fungal ASVs from *Aureobasidium*, *Alternaria*, *Neocucurbitaria*,* Exophiala*, *Penicillium*, *Filobasidium*, and *Cystobasidium* genera which were shared across the the three facilities throughout the two sampling seasons. To the best of our knowledge, no previous study has determined the environmental core microbiota of food processing facilities. Given that our results are limited to the three tree fruit packing facilities that were monitored in our study, we are unable to assess whether geographical location, temperature, and humidity of the processing plant, the food being manufactured, the soils introduced from farm environments, and/or the cleaning and sanitizing practices used within each facility affect the core microbiota.

### A subset of ASVs was indicative of *L. monocytogenes* contamination

In this study, we employed two methods (i.e., ALDEx2 and random forest) to detect bacterial and fungal ASVs that may be indicative of *L. monocytogenes’* contamination in tree fruit packing built environments. Specifically, the bacterial ASVs were members of the genera *Pseudomonas*, *Stenotrophomonas*, and* Microbacterium*, and the fungal ASVs were members of the taxonomic genera *Yarrowia*, *Kurtzmaniella*, *Cystobasidium*, *Paraphoma*, and *Cutaneotrichosporon*. *Pseudomonas* [97], *Flavobacterium* [73], and *Stenotrophomonas* [72, 98] are known biofilm formers that can attach to abiotic surfaces. This suggests that the presence of these taxa may support the persistence of *L. monocytogenes*, possibly through biofilm formation by physically protecting *L. monocytogenes* from the antimicrobial action of sanitizers. Previous studies that investigated interactions between *L. monocytogenes* and the taxa identified here have reported inconsistent results. Studies involving co-culturing of *Pseudomonas* spp. with *L. monocytogenes* have shown synergistic, neutral, or antagonistic effect of *Pseudomonas* on the survival of *L. monocytogenes*, depending on the strain and method used in a study [99, 100]. A co-culture experiment of *L. monocytogenes*, *L. innocua*, and Gram-negative microbiota including a strain of *Stenotrophomonas maltophila* showed partial inhibition of *L. monocytogenes* growth when compared to single cultures [101], suggesting that interactions between *L. monocytogenes* and *Stenotrophomonas* are species or strain specific. To the best of our knowledge, no studies have been conducted to determine potential interactions between *Listeria* spp. and *Microbacterium*, *Yarrowia*, *Kurtzmaniella*, *Cystobasidium*, *Paraphoma*, and *Cutaneotrichosporon*, indicating the need for further research to assess the effects of environmental microbiota on the survival and persistence of *L. monocytogenes* in food processing environments.

### Nanopore sequencing may be used for direct detection of *L. monocytogenes* DNA from environmental samples

Detection of pathogenic species, such as *L. monocytogenes*, using conventional culture-based methods is time-consuming and laborious. Shotgun metagenomics next-generation sequencing offers an alternative means of detection of pathogenic organisms directly from DNA extracted from environmental or clinical samples [102] and provides insight into microbiota composition. In clinical settings, Nanopore sequencing has been used to detect and identify infectious disease agents and antimicrobial resistance directly from clinical samples in hospitals, and to monitor disease transmission during the Zika and Ebola epidemics [102, 103]. In this study, we conducted a proof-of-concept experiment to assess the feasibility of using Nanopore long-read technology to detect the presence of *L. monocytogenes* DNA directly in DNA extracted from three environmental samples. All three sequenced samples were *L. monocytogenes*-positive, as determined by the enrichment method. However, *Listeria* spp. reads were not detected in these samples using 16S rRNA V4 amplicon sequencing. Using Nanopore sequencing, we detected *L. monocytogenes* reads in all three samples, and a few *Listeria* species reads, suggesting that *Listeria* DNA was present in the sampled environments. This demonstrates that by sequencing a single sample per flow cell, Nanopore sequencing allows for the detection of *L. monocytogenes* DNA in environmental samples. However, we did not quantify viable *L. monocytogenes* in these samples or evaluate the limit of detection. Detection of pathogens using metagenomic sequencing methods can be challenging if pathogen concentration in samples is low. For example, a previous study reported that 10^5^ CFUs of *E. coli* inoculated onto leafy greens samples were required to obtain pathogen-associated reads using metagenomic sequencing without an enrichment step [104]. Further, in our study, the detection of *Listeria* DNA did not provide information on whether the DNA originated from viable or dead cells, which is a major limitation of current metagenome-based direct detection methods [105]. Alternative methods that incorporate long-read sequencing of enrichments have been proposed for *L. monocytogenes* [106, 107] which could be applied to increase the speed and the sensitivity of detection, and to verify that *L. monocytogenes* DNA originates from living cells. Lastly, the cost of Nanopore shotgun sequencing is currently significantly (over 10 times) higher compared to traditional culture-based methods for the detection of *L. monocytogenes* from environmental samples using the approach reported in this study [108].

## Conclusions

Differences in the composition of microbiota among facilities over seasons suggest the need for spatial and temporal microbiota characterization to reliably identify core microbiota in food processing environments. While the total microbiota composition was not indicative of *L. monocytogenes* contamination in the monitored tree fruit packing facilities, we identified specific taxa that were informative for classification of samples into *L. monocytogenes*-positive and -negative categories. This suggests that they may facilitate the persistence of *L. monocytogenes* in the environment. Further research is needed to improve the understanding of the interactions between these taxa and *L. monocytogenes* and to characterize the underlying mechanisms by which these taxa may support the survival and growth of *L. monocytogenes*.

## Supplementary Information

**Additional file 1: Figure S1.** Number of 16S rRNA and ITS sequencing reads obtained in two sampling years. **Figure S2.** Bacterial and fungal microbiota composition in each facility and year. **Table S1.** Metadata for samples collected in year 2. **Table S2.** Differences in the occurrence of *L. monocytogenes* among samples collected from different facilities in year 2. **Table S3.** Differences in the occurrence of *L. monocytogenes* among facilities and sections between year 1 and 2. **Table S4.** Common and temporal core fungal ASVs that were present in all facilities throughout the two sampling seasons. **Table S5.** Network hubs for bacterial and fungal microbiota, identified as ASVs with the highest betweenness centrality.

## Data Availability

Amplicon sequences generated in Y1 are available under the BioProject PRJNA527988 [35]. Amplicon and Nanopore sequences generated in Y2 were deposited in NCBI SRA under the BioProject PRJNA660494. The datasets and scripts supporting the conclusions of this article are available in a GitHub repository at https://github.com/LauRolon/Apple-Year2. Supplementary figures and tables are available in a Word document accompanying the manuscript.

## Abbreviations

FDA: Food and Drug Administration
BAM: Bacteriological Analytical Manual
ASV: Amplicon Sequence Variant
PERMANOVA: Permutational Multivariate Analysis of Variance
CLR: Center-log ratio
