# Supplementary material for: The composition of environmental microbiota in three tree fruit packing facilities changed over seasons and contained taxa indicative of L. monocytogenes contamination
Source: Microbiome. 2023 Jun 5;11:128. doi: 10.1186/s40168-023-01544-8 (PMC10240739; doi:10.1186/s40168-023-01544-8)
Supplement: Supplementary file 2 — Additional file 1: Figure S1. Number of 16S rRNA and ITS sequencing reads obtained in two sampling years. Figure S2. Bacterial and fungal microbiota composition in each facility and year. Table S1. Metadata for samples collected in year 2. Table S2. Differences in the occurrence of L. monocytogenes among samples collected from different facilities in year 2. Table S3. Differences in the occurrence of L. monocytogenes among facilities and sections between year 1 and 2. Table S4. Common and temporal core fungal ASVs that were present in all facilities throughout the two sampling seasons. Table S5. Network hubs for bacterial and fungal microbiota, identified as ASVs with the highest betweenness centrality. [file 40168_2023_1544_MOESM1_ESM.docx]

**Supplementary Material**


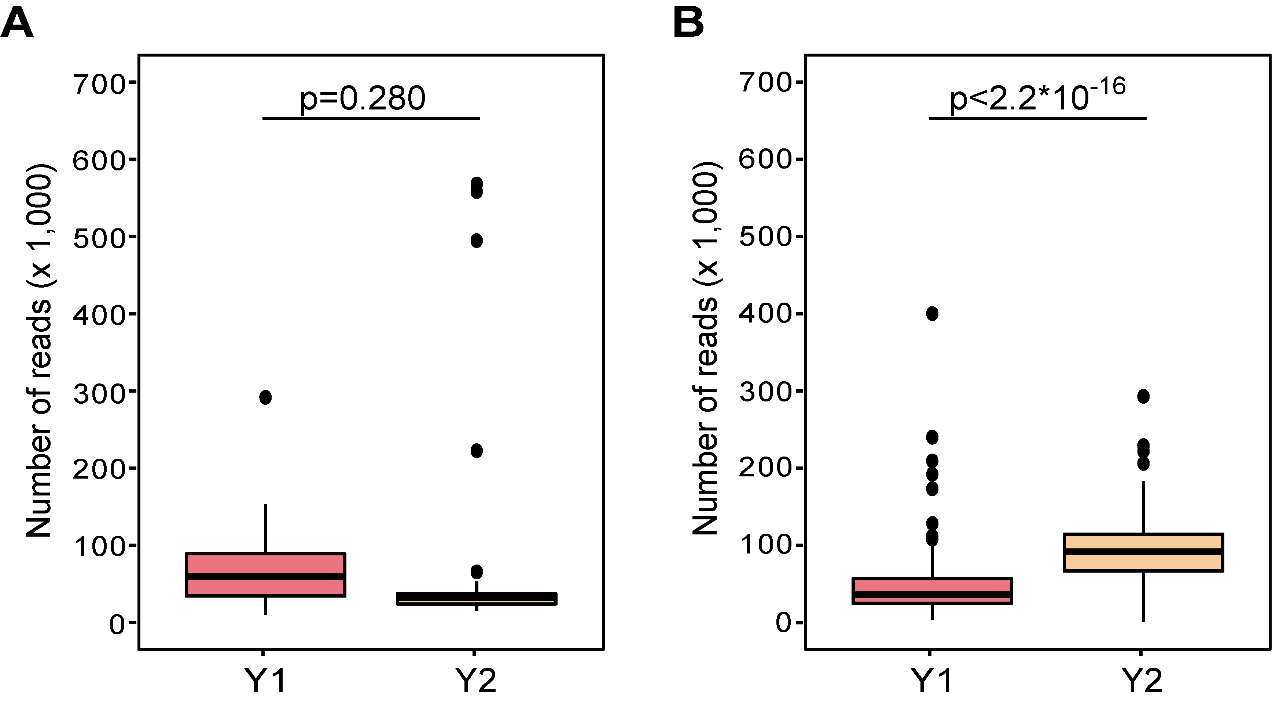


**Fig. S1: Number of 16S rRNA and ITS sequencing reads obtained in two sampling years**. Summary of the number of reads obtained from amplicon sequencing of the 16S rRNA V4 (A) and the ITS2 (B) in two sampling seasons (Y1 and Y2). The p values were obtained using a t-test by comparing means of reads between two sampling seasons.


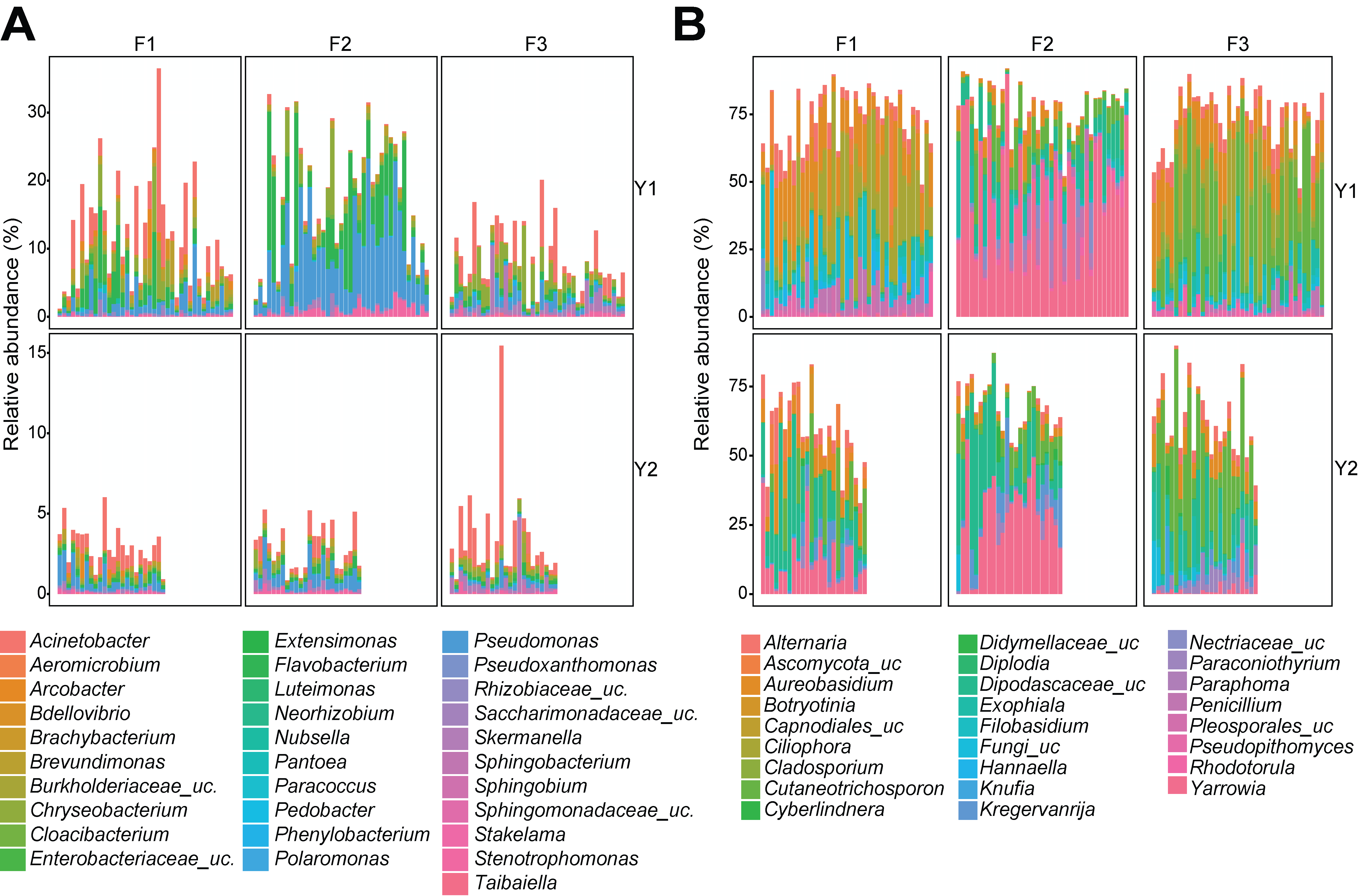


**Fig. S2:** **Bacterial and fungal microbiota composition in each facility and year.** Mean bacterial (A) and fungal (B) microbiota composition in each facility in year 1 (Y1) and year 2 (Y2) for all Amplicon Sequence Variants (ASVs) that had a relative abundance above 0.5% for bacteria or 10% for fungi in at least one sample. Each bar represents one sample, and they are presented in a chronological order of collection date on the x-axis. Bars are color-coded by taxonomic genus assigned to an ASV.

**Table S1:** Metadata for samples collected in year 2.

| **Bacterial microbiota sample name^a^** | **Bacteria microbiota BioSample accession number** | **Fungal microbiota sample** | **Fungal microbiota BioSample accession number** | **Facility** | **Section** | **Collection date** | **Collection week** | **Collection month** | ***L. monocytogenes* enrichment result** | **Metagenome**  **BioSample accession number** |
| --- | --- | --- | --- | --- | --- | --- | --- | --- | --- | --- |
| 1s-011119 | SAMN15951136 | 1t-011119 | SAMN15951243 | F1 | washing | 11-Jan-2019 | W05 | January | + |  |
| 1s-012519 | SAMN15951137 | 1t-012519 | SAMN15951244 | F1 | washing | 25-Jan-2019 | W06 | January | + |  |
| 1s-020719 | SAMN15951138 | 1t-020719 | SAMN15951245 | F1 | washing | 7-Feb-2019 | W07 | February | + |  |
| 1s-022119 (M1) | SAMN15951139 | 1t-022119 | SAMN15951246 | F1 | washing | 21-Feb-2019 | W08 | February | + | SAMN23587631 |
| 1s-110318 | SAMN15951144 | 1t-110318 | SAMN15951251 | F1 | washing | 3-Nov-2018 | W01 | November | + |  |
| 1s-112618 | SAMN15951145 | 1t-112618 | SAMN15951252 | F1 | washing | 26-Nov-2018 | W02 | November | - |  |
| 1s-120718 | SAMN15951146 | 1t-120718 | SAMN15951253 | F1 | washing | 7-Dec-2018 | W03 | December | + |  |
| 1s-121418 | SAMN15951147 | 1t-121418 | SAMN15951254 | F1 | washing | 14-Dec-2018 | W04 | December | - |  |
| 2s-011119 | SAMN15951148 | 2t-011119 | SAMN15951255 | F1 | drying | 11-Jan-2019 | W05 | January | - |  |
| 2s-012519 | SAMN15951149 | 2t-012519 | SAMN15951256 | F1 | drying | 25-Jan-2019 | W06 | January | + |  |
| 2s-020719 | SAMN15951150 | 2t-020719 | SAMN15951257 | F1 | drying | 7-Feb-2019 | W07 | February | + |  |
| 2s-022119 | SAMN15951151 | 2t-022119 | SAMN15951258 | F1 | drying | 21-Feb-2019 | W08 | February | - |  |
| 2s-110318 | SAMN15951156 | 2t-110318 | SAMN15951263 | F1 | drying | 3-Nov-2018 | W01 | November | - |  |
| 2s-112618 | SAMN15951157 | 2t-112618 | SAMN15951264 | F1 | drying | 26-Nov-2018 | W02 | November | - |  |
| 2s-120718 | SAMN15951158 | 2t-120718 | SAMN15951265 | F1 | drying | 7-Dec-2018 | W03 | December | - |  |
| 2s-121418 | SAMN15951159 | 2t-121418 | SAMN15951266 | F1 | drying | 14-Dec-2018 | W04 | December | - |  |
| 3s011119 | SAMN15951160 | 3t011119 | SAMN15951267 | F1 | waxing | 11-Jan-2019 | W05 | January | + |  |
| 3s012519 | SAMN15951161 | 3t012519 | SAMN15951268 | F1 | waxing | 25-Jan-2019 | W06 | January | - |  |
| 3s020719 | SAMN15951162 | 3t020719 | SAMN15951269 | F1 | waxing | 7-Feb-2019 | W07 | February | - |  |
| 3s022119 | SAMN15951163 | 3t022119 | SAMN15951270 | F1 | waxing | 21-Feb-2019 | W08 | February | + |  |
| 3s110318 | SAMN15951168 | 3t110318 | SAMN15951275 | F1 | waxing | 3-Nov-2018 | W01 | November | - |  |
| 3s112618 | SAMN15951169 | 3t112618 | SAMN15951276 | F1 | waxing | 26-Nov-2018 | W02 | November | + |  |
| 3s120718 | SAMN15951170 | 3t120718 | SAMN15951277 | F1 | waxing | 7-Dec-2018 | W03 | December | - |  |
| 3s121418 | SAMN15951171 | 3t121418 | SAMN15951278 | F1 | waxing | 14-Dec-2018 | W04 | December | - |  |
| 4s-011119 | SAMN15951172 | 4t-011119 | SAMN15951279 | F2 | washing | 11-Jan-2019 | W05 | January | + |  |
| 4s-012519 | SAMN15951173 | 4t-012519 | SAMN15951280 | F2 | washing | 25-Jan-2019 | W06 | January | + |  |
| 4s-020719 | SAMN15951174 | 4t-020719 | SAMN15951281 | F2 | washing | 7-Feb-2019 | W07 | February | + |  |
| 4s-022119 (M4) | SAMN15951175 | 4t-022119 | SAMN15951282 | F2 | washing | 21-Feb-2019 | W08 | February | + | SAMN23587632 |
| 4s-110318 | SAMN15951180 | 4t-110318 | SAMN15951287 | F2 | washing | 3-Nov-2018 | W01 | November | + |  |
| 4s-112618 | SAMN15951181 | 4t-112618 | SAMN15951288 | F2 | washing | 26-Nov-2018 | W02 | November | + |  |
| 4s-120718 | SAMN15951182 | 4t-120718 | SAMN15951289 | F2 | washing | 7-Dec-2018 | W03 | December | + |  |
| 4s-121418 | SAMN15951183 | 4t-121418 | SAMN15951290 | F2 | washing | 14-Dec-2018 | W04 | December | + |  |
| 5s-011119 | SAMN15951184 | 5t-011119 | SAMN15951291 | F2 | drying | 11-Jan-2019 | W05 | January | + |  |
| 5s-012519 | SAMN15951185 | 5t-012519 | SAMN15951292 | F2 | drying | 25-Jan-2019 | W06 | January | + |  |
| 5s-110318 | SAMN15951186 | 5t-110318 | SAMN15951293 | F2 | drying | 3-Nov-2018 | W01 | November | + |  |
| 5s-112618 | SAMN15951187 | 5t-112618 | SAMN15951294 | F2 | drying | 26-Nov-2018 | W02 | November | + |  |
| 5s-120718 | SAMN15951191 | 5t-120718 | SAMN15951298 | F2 | drying | 7-Dec-2018 | W03 | December | + |  |
| 5s-121418 | SAMN15951192 | 5t-121418 | SAMN15951299 | F2 | drying | 14-Dec-2018 | W04 | December | + |  |
| 6s-011119 | SAMN15951193 | 6t-011119 | SAMN15951300 | F2 | waxing | 11-Jan-2019 | W05 | January | + |  |
| 6s-012519 | SAMN15951194 | 6t-012519 | SAMN15951301 | F2 | waxing | 25-Jan-2019 | W06 | January | + |  |
| 6s-020719 | SAMN15951195 | 6t-020719 | SAMN15951302 | F2 | waxing | 7-Feb-2019 | W07 | February | + |  |
| 6s-022119 | SAMN15951196 | 6t-022119 | SAMN15951303 | F2 | waxing | 21-Feb-2019 | W08 | February | + |  |
| 6s-110318 | SAMN15951197 | 6t-110318 | SAMN15951304 | F2 | waxing | 3-Nov-2018 | W01 | November | + |  |
| 6s-112618 | SAMN15951198 | 6t-112618 | SAMN15951305 | F2 | waxing | 26-Nov-2018 | W02 | November | + |  |
| 6s-120718 | SAMN15951203 | 6t-120718 | SAMN15951310 | F2 | waxing | 7-Dec-2018 | W03 | December | + |  |
| 6s-121418 | SAMN15951204 | 6t-121418 | SAMN15951311 | F2 | waxing | 14-Dec-2018 | W04 | December | + |  |
| 7s-011119 | SAMN15951205 | 7t-011119 | SAMN15951312 | F3 | washing | 11-Jan-2019 | W05 | January | + |  |
| 7s-012519 | SAMN15951206 | 7t-012519 | SAMN15951313 | F3 | washing | 25-Jan-2019 | W06 | January | + |  |
| 7s-020719 | SAMN15951207 | 7t-020719 | SAMN15951314 | F3 | washing | 7-Feb-2019 | W07 | February | + |  |
| 7s-022119 (M7) | SAMN15951208 | 7t-022119 | SAMN15951315 | F3 | washing | 21-Feb-2019 | W08 | February | + | SAMN23587633 |
| 7s-110318 | SAMN15951209 | 7t-110318 | SAMN15951316 | F3 | washing | 3-Nov-2018 | W01 | November | - |  |
| 7s-112618 | SAMN15951210 | 7t-112618 | SAMN15951317 | F3 | washing | 26-Nov-2018 | W02 | November | + |  |
| 7s-120718 | SAMN15951215 | 7t-120718 | SAMN15951322 | F3 | washing | 7-Dec-2018 | W03 | December | + |  |
| 7s-121418 | SAMN15951216 | 7t-121418 | SAMN15951323 | F3 | washing | 14-Dec-2018 | W04 | December | + |  |
| 8s-011119 | SAMN15951217 | 8t-011119 | SAMN15951324 | F3 | drying | 11-Jan-2019 | W05 | January | + |  |
| 8s-012519 | SAMN15951218 | 8t-012519 | SAMN15951325 | F3 | drying | 25-Jan-2019 | W06 | January | + |  |
| 8s-020719 | SAMN15951219 | 8t-020719 | SAMN15951326 | F3 | drying | 7-Feb-2019 | W07 | February | + |  |
| 8s-022119 | SAMN15951220 | 8t-022119 | SAMN15951327 | F3 | drying | 21-Feb-2019 | W08 | February | + |  |
| 8s-110318 | SAMN15951221 | 8t-110318 | SAMN15951328 | F3 | drying | 3-Nov-2018 | W01 | November | + |  |
| 8s-112618 | SAMN15951222 | 8t-112618 | SAMN15951329 | F3 | drying | 26-Nov-2018 | W02 | November | + |  |
| 8s-120718 | SAMN15951227 | 8t-120718 | SAMN15951334 | F3 | drying | 7-Dec-2018 | W03 | December | + |  |
| 8s-121418 | SAMN15951228 | 8t-121418 | SAMN15951335 | F3 | drying | 14-Dec-2018 | W04 | December | + |  |
| 9s-011119 | SAMN15951229 | 9t-011119 | SAMN15951336 | F3 | waxing | 11-Jan-2019 | W05 | January | + |  |
| 9s-012519 | SAMN15951230 | 9t-012519 | SAMN15951337 | F3 | waxing | 25-Jan-2019 | W06 | January | + |  |
| 9s-020719 | SAMN15951231 | 9t-020719 | SAMN15951338 | F3 | waxing | 7-Feb-2019 | W07 | February | + |  |
| 9s-022119 | SAMN15951232 | 9t-022119 | SAMN15951339 | F3 | waxing | 21-Feb-2019 | W08 | February | + |  |
| 9s-110318 | SAMN15951233 | 9t-110318 | SAMN15951340 | F3 | waxing | 3-Nov-2018 | W01 | November | - |  |
| 9s-112618 | SAMN15951234 | 9t-112618 | SAMN15951341 | F3 | waxing | 26-Nov-2018 | W02 | November | + |  |
| 9s-120718 | SAMN15951239 | 9t-120718 | SAMN15951346 | F3 | waxing | 7-Dec-2018 | W03 | December | + |  |
| 9s-121418 | SAMN15951240 | 9t-121418 | SAMN15951347 | F3 | waxing | 14-Dec-2018 | W04 | December | + |  |

^a^Sample names in parentheses correspond to the ID used for Nanopore sequencing.

**Table S2:** Differences in the occurrence of *L. monocytogenes* among samples collected from different facilities in year 2.

| *L. monocytogenes* detection | Facility | | |
| --- | --- | --- | --- |
|  | F1 | F2 | F3 |
| Absent | 13 | 0 | 2 |
| Present | 11 | 24 | 22 |
|  | Chi-square | DF^a^ | P-value^b^ |
| Pearson | 26.82 | 2 | 1.50*10^-6^ |
| Likelihood | 24.76 | 2 | 4.21*10^-6^ |

^a^DF, degrees of freedom.

^b^Chi-square test was carried out, followed by pairwise comparisons using Fisher’s Exact test with Bonferroni correction for multiple comparisons.

**Table S3:** Differences in the occurrence of *L. monocytogenes* among facilities and sections between year 1 and 2.

| **Variable** | **Level** | **χ^2^** | **DF^a^** | **p-value** |
| --- | --- | --- | --- | --- |
| Facility^b^ | F1 | 1.330 | 1 | 0.25 |
|  | F3 | 13.873 | 1 | 1.96*10^-4^ |
| Section | Washing | 1.819 | 1 | 0.18 |
|  | Drying | 1.996 | 1 | 0.16 |
|  | Waxing | 3.939 | 1 | 0.047 |

^a^DF, degrees of freedom.

^b^F2 was not included in this analysis as all the samples collected in this facility were positive for *L. monocytogenes* in both seasons.

**Table S4:** Common and temporal core fungal ASVs that were present in all facilities throughout the two sampling seasons.

| **ASV^a^** | **Kingdom** | **Phylum** | **Class** | | **Order** | | **Family** | | **Genus** | | | **Species** | |
| --- | --- | --- | --- | --- | --- | --- | --- | --- | --- | --- | --- | --- | --- |
| 2 | Fungi | Ascomycota | | *Dothideomycetes* | | *Dothideales* | | *Aureobasidiaceae* | | *Aureobasidium* | *Pullulans* | |  |
| 6 |  |  |  |  |  | *Pleosporales* | | *Pleosporaceae* | | *Alternaria* | *Betar-kenyensis* | |  |
| 21 |  |  |  |  |  |  |  | *Cucurbitariaceae* | | *Neocucurbitaria* | *Neocucurbitaria_unclassified* | |  |
| 9 |  |  |  | *Eurotiomycetes* | | *Chaetothyriales* | | *Herpotrichiellaceae* | | *Exophiala* | *Exophiala_unclassified* | |  |
| 10 |  |  |  |  |  |  |  |  |  |  | *Cancerae* | |  |
| 13 |  |  |  |  |  | *Eurotiales* | | *Aspergillaceae* | | *Penicillum* | *Aurantiogriseum* | |  |
| 17 |  | Basidiomycota | | *Tremellomycetes* | | *Filobasidiales* | | *Filobasidiaceae* | | *Filobasidium* | *Floriforme* | |  |
| 18 |  |  |  | *Cystobasidiomycetes* | | *Cystobacidiales* | | *Cystobasidiaceae* | | *Cystobasidium* | *Slooffiae* | |  |

^a^ASV, exact sequence variant.

**Table S5:** Network hubs for bacterial and fungal microbiota, identified as ASVs with the highest betweenness centrality.

| **ASV^a^** | **Kingdom** | **Phylum** | **Class** | **Order** | **Family** | **Genus^b^** | **Species^b^** |
| --- | --- | --- | --- | --- | --- | --- | --- |
| 1016 | Bacteria | Actinobacteria | *Actinobacteria* | *Corynebacteriales* | *Nocardiaceae* | *Rhodococcus* | *Rhodococcus_uc.* |
| 570 |  |  |  |  |  | *Williamsia* | *Williamsia_uc.* |
| 529 |  |  |  | *Micrococcales* | *Microbacteriaceae* | *Microbacterium* | *Microbacterium_uc.* |
| 1920 |  |  |  |  |  |  |  |
| 1870 |  |  |  |  |  | *Pseudoclavibacter* | *Pseudoclavibacter_uc.* |
| 163 |  |  |  |  | *Micrococcaceae* | *Kocuria* | *Kocuria_uc.* |
| 767 |  | Bacteroidetes | *Bacteroidia* | *Flavobacteriales* | *Flavobacteriaceae* | *Flavobacterium* | *Flavobacterium_uc.* |
| 1065 |  |  |  |  |  |  |  |
| 1568 |  |  |  |  |  |  |  |
| 2513 |  |  |  |  |  |  |  |
| 1126 |  |  |  |  | *Weeksellaceae* | *Chryseobacterium* | *Chryseobacterium_uc.* |
| 1259 |  |  |  |  |  |  |  |
| 1531 |  |  |  |  |  |  |  |
| 1939 |  |  |  |  |  |  |  |
| 1049 |  |  |  |  |  | *Cloacibacterium* | *Cloacibacterium_uc.* |
| 558 |  |  |  | *Sphingobacteriales* | *Sphingobacteriaceae* | *Pedobacter* | *Pedobacter_uc.* |
| 980 |  |  |  |  |  |  |  |
| 1109 |  |  |  |  |  |  |  |
| 1644 |  | Patescibacteria | *Saccharimonadia* | *Saccharimonadales* | *Saccharimonadales_uc.* | *Saccharimonadales_uc.* | *Saccharimonadales_uc.* |
| 2089 |  |  |  |  |  |  |  |
| 2179 |  |  |  |  |  |  |  |
| 385 |  | Proteobacteria | *Alphaproteobacteria* | *Caulobacterales* | *Caulobacteraceae* | *Brevundimonas* | *Brevundimonas_uc.* |
| 514 |  |  |  |  |  |  |  |
| 544 |  |  |  |  |  |  |  |
| 1118 |  |  |  |  |  |  |  |
| 1270 |  |  |  |  |  |  |  |
| 1335 |  |  |  |  |  |  |  |
| 1434 |  |  |  |  |  |  |  |
| 586 | Bacteria | Proteobacteria | *Alphaproteobacteria* | *Rhizobiales* | *Beijerinckiaceae* | *Bosea* | *Bosea_uc.* |
| 329 |  |  |  |  | *Rhizobiaceae* | *Allorhizobium-Neorhizobium-Pararhizobium-Rhizobium* | *Allorhizobium-Neorhizobium-Pararhizobium-Rhizobium_uc.* |
| 592 |  |  |  |  |  |  |  |
| 712 |  |  |  |  |  |  |  |
| 721 |  |  |  |  |  |  |  |
| 1523 |  |  |  |  |  |  |  |
| 2510 |  |  |  |  |  |  |  |
| 1748 |  |  |  |  | *Rhizobiaceae* | *Rhizobiaceae_uc.* | *Rhizobiaceae_uc.* |
| 1616 |  |  |  | *Rhodobacterales* | *Rhodobacteraceae* | *Haematobacter* | *Haematobacter_uc.* |
| 3150 |  |  |  |  |  |  |  |
| 367 |  |  |  |  |  | *Paracoccus* | *Paracoccus_uc.* |
| 1298 |  |  |  |  |  |  |  |
| 571 |  |  |  |  |  | *Rhodobacteraceae_uc.* | *Rhodobacteraceae_uc.* |
| 121 |  |  |  | *Sphingomonadales* | *Sphingomonadaceae* | *Sphingobium* | *Sphingobium_uc.* |
| 387 |  |  |  |  |  |  |  |
| 472 |  |  |  |  |  |  |  |
| 476 |  |  |  |  |  |  |  |
| 1096 |  |  |  |  |  |  |  |
| 1167 |  |  |  |  |  |  |  |
| 1277 |  |  |  |  |  |  |  |
| 1294 |  |  |  |  |  |  |  |
| 1063 |  |  |  |  |  | *Sphingomonadaceae_uc.* | *Sphingomonadaceae_uc.* |
| 313 |  |  |  |  |  | *Sphingomonas* | *Sphingomonas_uc.* |
| 728 |  |  |  |  |  |  |  |
| 886 |  |  |  |  |  |  |  |
| 1628 |  |  |  |  |  |  |  |
| 2376 |  |  |  |  |  | *Sphingopyxis* | *Sphingopyxis_uc.* |
| 9158 |  |  |  |  |  |  |  |
| 2319 |  |  |  |  |  | *Stakelama* | *Stakelama_uc.* |
| 1105 |  |  | *Deltaproteobacteria* | *Bdellovibrionales* | *Bdellovibrionaceae* | *Bdellovibrio* | *Bdellovibrio_uc.* |
| 1206 | Bacteria | Proteobacteria | *Deltaproteobacteria* | *Bdellovibrionales* | *Bdellovibrionaceae* | *Bdellovibrio* | *Bdellovibrio_uc.* |
| 1330 |  |  |  |  |  |  |  |
| 2694 |  |  |  |  |  |  |  |
| 1050 |  |  | *Gammaproteobacteria* | *Betaproteobacteriales* | *Burkholderiaceae* | *Acidovorax* | *Acidovorax_uc.* |
| 419 |  |  |  |  |  | *Burkholderiaceae_uc.* | *Burkholderiaceae_uc.* |
| 450 |  |  |  |  |  |  |  |
| 531 |  |  |  |  |  |  |  |
| 819 |  |  |  |  |  |  |  |
| 1193 |  |  |  |  |  |  |  |
| 1876 |  |  |  |  |  |  |  |
| 2575 |  |  |  |  |  |  |  |
| 1702 |  |  |  |  |  | *Delftia* | *Delftia_uc.* |
| 1451 |  |  |  |  |  | *Variovorax* | *Variovorax_uc.* |
| 1609 |  |  |  |  |  | *Xenophilus* | *Xenophilus_uc.* |
| 466 |  |  |  | *Enterobacteriales* | *Enterobacteriaceae* | *Enterobacteriaceae_uc.* | *Enterobacteriaceae_uc.* |
| 812 |  |  |  |  |  |  |  |
| 263 |  |  |  | *Pseudomonadales* | *Pseudomonadaceae* | *Pseudomonas* | *Pseudomonas_uc.* |
| 810 |  |  |  |  |  |  |  |
| 1810 |  |  |  |  |  |  |  |
| 1837 |  |  |  |  |  |  |  |
| 1881 |  |  |  |  |  |  |  |
| 2377 |  |  |  |  |  |  |  |
| 3728 |  |  |  |  |  |  |  |
| 9459 |  |  |  |  |  |  |  |
| 590 |  |  |  | *Xanthomonadales* | *Xanthomonadaceae* | *Pseudoxanthomonas* | *Pseudoxanthomonas_uc.* |
| 180 |  |  |  |  |  | *Stenotrophomonas* | *Stenotrophomonas_uc.* |
| 495 |  |  |  |  |  |  |  |
| 1627 |  |  |  |  |  |  |  |
| 1809 |  |  |  |  |  |  |  |
| 4635 |  |  |  |  |  |  |  |
| 3659 | Bacteria | Proteobacteria | *Gammaproteobacteria* | *Xanthomonadales* | *Xanthomonadaceae* | *Thermomonas* | *Thermomonas_uc.* |
| 8 | Fungi | Ascomycota | *Dothideomycetes* | *Pleosporales* | *Cucurbitariaceae* | *Neocucurbitaria* | *keratinophila* |
| 11 |  |  |  |  | *Didymosphaeriaceae* | *Paraconiothyrium* | *Paraconiothyrium_uc.* |
| 12 |  |  |  |  | *Phaeosphaeriaceae* | *Muriphaeosphaeria* | *viburni* |
| 22 |  |  |  |  |  | *Neosetophoma* | *samararum* |
| 23 |  |  | *Eurotiomycetes* | *Chaetothyriales* | *Cyphellophoraceae* | *Cyphellophora* | *pluriseptata* |
| 24 |  |  | *Leotiomycetes* | *Helotiales* | *Vibrisseaceae* | *Phialocephala* | *Phialocephala_uc.* |
| 28 |  |  | *Saccharomycetes* | *Saccharomycetales* | *Pichiaceae* | *Kregervanrija* | *fluxuum* |
| 36 |  |  |  | *Saccharomycetales* | *Saccharomycetales_fam_Incertae_sedis* | *Candida* | *incommunis* |
| 41 |  | Basidiomycota | *Cystobasidiomycetes* | *Cystobasidiales* | *Cystobasidiaceae* | *Cystobasidium* | *pinicola* |
| 45 |  |  | *Tremellomycetes* | *Tremellales* | *Bulleraceae* | *Genolevuria* | *Genolevuria_uc.* |
| 54 |  |  |  |  | *Bulleribasidiaceae* | *Vishniacozyma* | *carnescens* |
| 65 |  |  |  |  |  |  | *foliicola* |
| 83 |  |  |  |  |  |  | *heimaeyensis* |
| 94 |  |  |  |  |  |  | *Vishniacozyma_uc.* |
| 116 |  |  |  | *Trichosporonales* | *Trichosporonaceae* | *Cutaneotrichosporon* | *jirovecii* |
| 139 |  |  |  |  |  |  | *moniliiforme* |
| 169 |  |  |  |  |  | *Trichosporonaceae_uc.* | *Trichosporonaceae_uc.* |
| 250 |  | Fungi_uc. | *Fungi_uc.* | *Fungi_uc.* | *Fungi_uc.* | *Fungi_uc.* | *Fungi_uc.* |

^a^ASV, exact sequence variant.

^b^Uc.: unclassified
